# Mesenchymal stem cells receive adaptive islet–derived *miR-151*–containing sEVs to promote β cell compensation in obesity

**DOI:** 10.1126/sciadv.adu4196

**Published:** 2026-07-17

**Authors:** Xinwei Guo, Yang Wang, Ruixue Du, Wei Yong, Wenjing Yan, Zicheng Zhang, Yi Pan, Yanfeng Zhang, Yumeng Shen, Yue Yang, Fangfang Zhang, Jianxing Liu, Wei Tang, Yue Liu, Liang Jin

**Affiliations:** ^1^ State Key Laboratory of Natural Medicines, Jiangsu Key Laboratory of Druggability of Biopharmaceuticals, School of Life Science and Technology, China Pharmaceutical University, 24 Tongjiaxiang, Nanjing, Jiangsu, China.; ^2^ Department of Endocrinology, Geriatric Hospital of Nanjing Medical University, 30 Luojia Road, Nanjing, Jiangsu, China.; ^3^ School of Pharmacy, Ningxia Medical University, Yinchuan, Ningxia Hui Autonomous Region, China.

## Abstract

Pancreatic islets respond to obesity-related insulin resistance by increasing β cell mass and insulin secretion. However, the molecular mechanisms behind this vital compensation are not fully understood. This study shows that adaptive islet–derived small extracellular vesicles (aid-sEVs) play a key role in β cell adaptation in obesity. Aid-sEV production rises under hyperlipidemic conditions, and uptake by adjacent cells occurs via F11R-mediated recognition. Mesenchymal stem cells (MSCs) act as downstream effectors after they internalize aid-sEVs, promoting β cell–adaptive responses. These vesicles deliver *miR-151* to MSCs, triggering *miR-151–*dependent cellular reprogramming toward a Wnt-secreting phenotype. Restoration of *miR-151* in microRNA-deficient aid-sEVs restores their proadaptive effects on β cells. *Klf9*, a direct target of *miR-151*, is involved in regulating MSC proliferation and WNT secretion by controlling *Wnt3a* and *Ccnd1* transcription. These findings reveal a critical pathway controlling β cell compensation in diet-induced obesity and indicate that targeted enhancement of aid-sEV secretion could be a therapeutic strategy to counteract β cell dysfunction in diabetic patients.

## Teaser

Mesenchymal stem cell uptake of islet-derived *miR-151–*enriched sEVs improves β cell compensation in obesity.

## INTRODUCTION

Type 2 diabetes (T2D) is a complex metabolic disorder posing a notable global health burden due to its rising prevalence and long-term complications. Obesity is a key risk factor for T2D, closely associated with insulin resistance, dyslipidemia, and progressive impairment of glucose homeostasis. In response to increased metabolic demand, pancreatic β cells undergo adaptive changes, including expansion of β cell mass and up-regulation of insulin biosynthesis and secretion, thus preserving normoglycemia despite reduced insulin sensitivity (*1*). If this adaptive β cell expansion fails, then insulin production becomes insufficient, leading to the development of overt T2D (*2*). Therapeutic strategies that enhance β cell replication and maintain functional β cell mass have therefore been proposed as viable approaches to delaying disease progression or restoring metabolic control in T2D mellitus (*3*). Therefore, a comprehensive understanding of the mechanisms underlying β cell compensation in obesity is crucial for developing effective targeted antidiabetic treatments.

Small extracellular vesicles (sEVs) are nanosized, lipid bilayer–enclosed vesicles typically 30 to 150 nm in diameter, constitutively released by most cell types (*4*). These vesicles facilitate intercellular communication by transporting a diverse repertoire of bioactive molecules (proteins, lipids, and nucleic acids), with microRNAs (miRNAs) serving as key posttranscriptional regulators (*5*). sEVs exert biological effects both locally within tissue microenvironments and systemically after entering the circulation, thus mediating prolonged signaling between metabolically active organs (*6*). Despite increasing recognition of sEV-mediated regulatory networks in metabolic diseases, the molecular composition, cellular targets, and functional significance of pancreatic islet–derived sEVs under obese conditions remain incompletely defined. Previous studies have shown that sEVs released from islets during early-stage obesity modulate hepatic insulin sensitivity and whole-body glucose homeostasis in recipient mice, primarily through *miR-29* family members (*7*). Inhibition of exosomal *miR-155* derived from islet-resident macrophages has been shown to ameliorate insulin resistance and glucose intolerance in high-fat diet (HFD) feeding mice (*8*). In addition, islet-derived exosomal *miR-204* mediates islet–skeletal muscle communication in obesity (*9*). Given that the sEV production is influenced by nutritional availability, we hypothesize that pancreatic islet–derived sEVs may undergo acute functional changes in receptor cells to regulate β cell compensation.

Mesenchymal stem cells (MSCs) are present in almost all tissues and are crucial for tissue regeneration and homeostasis (*10*). Infusion of MSCs into diabetic mice improves blood glucose levels and promotes β cell regeneration (*11*). Research conducted by Bhansali *et al.* (*12*) and Liu *et al.* (*13*) indicated that treatment with MSCs improved metabolic control and β cell function in patients with T2D. Immunomodulation or transdifferentiation is often used as a possible mechanism to explain the above effects (*14*, *15*). Recent research has shown that MSC-derived exosomes can reverse peripheral insulin resistance and relieve cellular destruction (*16*), suggesting a cross-talk between MSCs and islets. Further investigation is needed to determine whether MSCs can regulate β cell compensation in obesity by communicating with pancreatic islets.

In this study, we found that as obesity progresses, the islet-derived sEVs are elevated, and the number of MSCs in the pancreatic islet also increases. The islet β cell transfers *miR-151–*containing sEVs to MSCs in vivo, which reciprocate by releasing Wnts to β cells. Adaptive islet–derived sEV (aid-sEVs) are “self-rescue” signals for β cells to initiate compensation in obesity. Understanding the molecular communication between β cells and MSCs will facilitate the development of more effective strategies for preventing T2D.

## RESULTS

### The sEV secretion is elevated in adaptive islet β cells

To explore the role of sEVs in the development of islet adaptation, we fed the wild-type C57BL/6J mice with increasing duration of HFD to recapitulate features of β cell compensation (fig. S1A). The body weight and blood glucose were increased in mice fed HFD relative to the normal chow diet (NCD)–fed mice (fig. S1, B and C). As the feeding time of HFD increased, mice gradually showed glucose intolerance (fig. S1, D to G), hyperinsulinemia (fig. S1H), and hyperlipidemia (fig. S1, I and J). In addition, we found a notable enhancement in glucose-stimulated insulin secretion (GSIS) in islets isolated from HFD-fed mice compared to mice fed the NCD (fig. S1, K to N). Consistent with this, mice fed with HFD for 9 weeks showed an increase in pancreatic islet size and notable proliferation of β cell (fig. S1, O and P). This indicates that HFD feeding can recapitulate features of adaptation, and we chose mice fed with HFD for 9 weeks to represent the islet β cell compensatory phases in subsequent experiments.

**Fig. 1.**
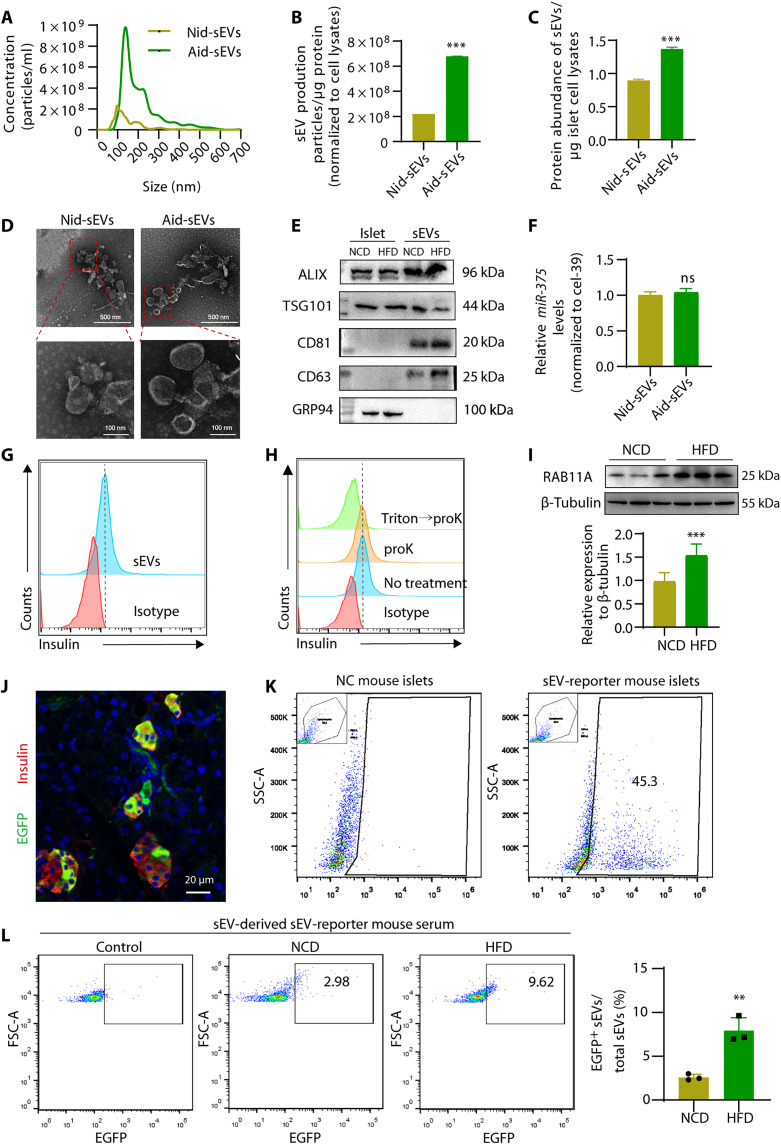
The sEV secretion is elevated in adaptive islet β cell. (**A**) NTA of nid-sEVs and aid-sEVs (*n* = 3 per group). (**B**) NTA results of nid-sEVs and aid-sEVs were normalized by cell lysates of donor cells. The islets of normal and compensatory mice were lysed, and the total protein concentration of the lysates was determined by BCA assay. The particle concentration obtained from NTA was then normalized to the total cellular protein amount from the corresponding donor cells (*n* = 3 per group). (**C**) Protein abundance of nid-sEVs and aid-sEVs was normalized by cell lysates of donor cells (*n* = 3 per group). (**D**) TEM images of nid-sEVs and aid-sEVs. Scale bars, 500 and 100 nm. (**E**) Western blot of sEV markers in nid-sEVs and aid-sEVs (*n* = 3). (**F**) Quantitative reverse transcription polymerase chain reaction (qRT-PCR) was conducted to detect the expression level of *miR-375* in nid-sEVs and aid-sEVs (*n* = 3 per group). (**G** and **H**) The sEVs were attached to 4-μm aldehyde/sulfate latex beads and stained with insulin (G). The sEV-bound beads were treated with proteinase K (proK) or Triton X-100. Flow cytometry was applied to detect the insulin inside the sEVs (H). (**I**) Western blot of RAB11A in islets of NCD and HFD mice (*n* = 3 per group). (**J** and **K**) After 4 weeks of Ins2-CD63-EGFP-AAV injection, pancreases were obtained and stained with insulin (red) and EGFP (green). Magnification, ×20; scale bar, 20 μm (J). Islets were isolated and digested into single-cell suspension. Cells were analyzed by flow cytometry to detect EGFP^+^ signal. Negative control (NC) mice islets were used as control (K). *n* = 3 mice per group. (**L**) Serum were collected from Ins2-CD63-EGFP-AAV–injected NCD and HFD mice. sEVs were isolated and attached to 4-μm aldehyde/sulfate latex beads. Flow cytometry was applied to detect the EGFP^+^ signal in the sEV-bound beads (*n* = 3 per group). Data are presented as means ± SD. *P < 0.05, **P < 0.01, and ***P < 0.001 by Student’s *t* test [(B), (C), (F), (I), and (L)]. ns, not significant.

We next sought to determine whether there were differences in the concentration and size of sEVs secreted by the islets of normal and compensatory mice (normal islet–derived sEVs herewith referred to as nid-sEVs, while adaptive islet-derived sEVs are referred to as aid-sEVs). The islets were isolated and incubated in an oxygenated, nutrient-rich buffer, as described previously by our laboratory (*17*–*19*). Islet-secreted factors were collected after 48-hour incubation, and sEVs were isolated and purified by ultracentrifugation. Nanoparticle tracking analysis (NTA) showed that most of the sEVs were 50 to 150 nm and were present at a markedly higher concentration in adaptive islets than in normal islets (Fig. 1A). Through normalized particle numbers by cell lysates, we further confirmed that adaptive mice release higher levels of sEVs (Fig. 1B). Protein quantification of sEVs also corroborated this finding (Fig. 1C). Transmission electron microscopy (TEM) analysis of nid-sEVs and aid-sEVs demonstrated the compatible sizes and shapes (Fig. 1D). The presence of validated sEV proteins, including ALIX, TSG101, CD63, and CD81, was confirmed using Western blot analysis (Fig. 1E). Endoplasmic reticulum–related Grp94, a negative marker of sEVs, was detected in protein samples of islet but was undetectable in sEVs (Fig. 1E). Moreover, the β cell–specific insulin and *miR-375* were also detected in the sEVs (Fig. 1, F and G). Further validation was conducted using Triton X-100 and proteinase K digestion, which confirmed that insulin was encapsulated within sEVs rather than representing extracellular contamination (Fig. 1H). Expression of RAB11A, a regulator of sEV biogenesis (*20*), was increased in adaptive islets (Fig. 1I). These results indicate that sEVs originate predominantly from β cells and that adaptive islet compensation is associated with enhanced sEV secretion without altering vesicle size or canonical marker expression.

To examine whether β cell–derived sEV secretion was higher in vivo, this study developed an adeno-associated virus (AAV)–based reporter system. The construct encoded CD63 fused to enhanced green fluorescent protein (EGFP) under the control of the β cell–specific *insulin 2* (*Ins2*) promoter, allowing selective labeling of β cell–derived sEVs (fig. S1Q). Ins2-CD63-EGFP-AAV vectors were injected via pancreatic ductal infusion into 8-week-old C57BL/6J mice (fig. S1R). Positive EGFP expression was observed in pancreatic islets 4 weeks postinjection, confirming successful reporter expression in vivo (Fig. 1, J and K). Although Ins2-CD63-EGFP-AAV was only injected into the pancreas, we could detect the existence of EGFP^+^ sEVs in the serum (Fig. 1L). We also found that patients with hypercholesterolemia (total cholesterol > 5.2 mM) and hypertriglyceride (triglyceride > 1.7 mM) have higher levels of sEVs in their blood (fig. S1, S and T) and serum sEV levels are positively correlated with serum total cholesterol level and triglyceride (fig. S1, U and V). Despite the fact that it cannot be proven that sEVs in human serum are derived from pancreatic islets, combined with animal data, we hypothesize that higher levels of sEVs in obese individuals may be related to β cell compensation.

### Aid-sEVs promote the development of islet β cell compensation

**Fig. 2.**
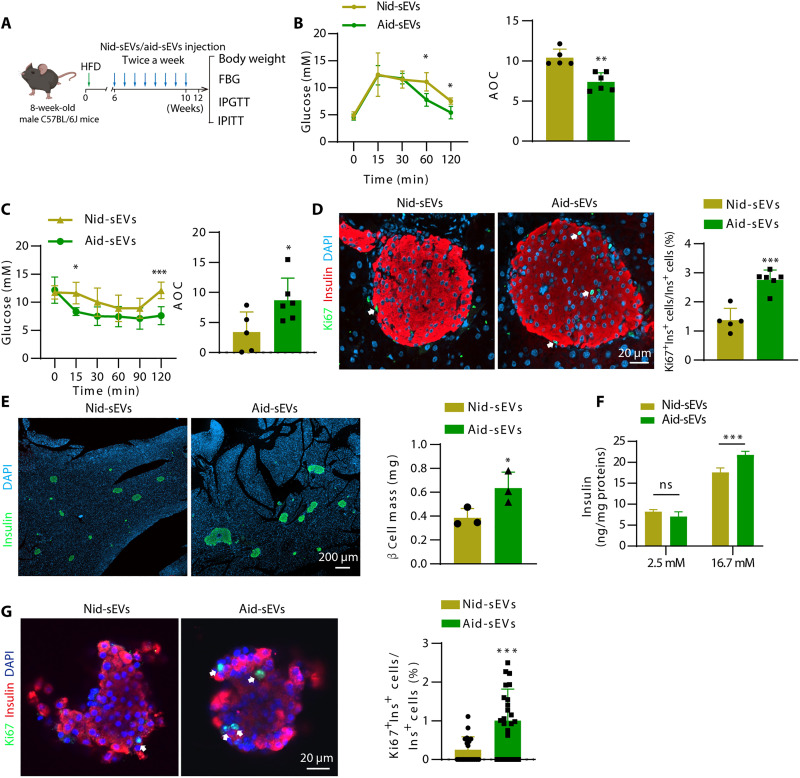
Aid-sEVs promote the development of islet β cell compensation. (**A**) The flowchart of the in vivo experiment designed for detecting the function of aid-sEVs on β cell. Eight-week-old C57BL/6J mice were fed with an HFD diet for 6 weeks and administered with nid-sEVs or aid-sEVs for 4 weeks by tail vein injection (30 μg per mice, twice a week). The icon or graphical images were created in BioRender. Guo, X. (2026) https://BioRender.com/bw6f83d. (**B** and **C**) IPGTT (B) and IPITT (C) were performed in mice injected with nid-sEVs and aid-sEVs. The area of curve (AOC) of blood glucose level was calculated (*n* ≥ 5 mice per group). (**D**) Representative images and statistical analysis of Ki67^+^ β cells in mice injected with nid-sEVs and aid-sEVs (*n* ≥ 5 mice per group). Magnification, ×20; scale bar, 20 μm. DAPI, 4′,6-diamidino-2-phenylindole. (**E**) Representative images and statistical analysis of islet mass in mice injected with nid-sEVs and aid-sEVs (*n* = 3 mice per group). Magnification, ×4; scale bar, 200 μm. (**F**) Primary islets were isolated and treated with nid-sEVs or aid-sEVs for 24 hours. GSIS assay was performed on these islets (*n* = 3 per group). (**G**) Primary islets were isolated and treated with nid-sEVs or aid-sEVs for 24 hours, and then the islets were stained with Ki67 (green) and insulin (red). At least 20 islets were analyzed in each group. Magnification, ×20; scale bar, 20 μm. Data are presented as means ± SD. *P < 0.05, **P < 0.01, and ***P < 0.001 by Student’s *t* test [(D) to (G)] or two-way analysis of variance (ANOVA) [(B) and (C)].

Given that sEVs are pronouncedly elevated in adaptive mice, we then attempt to explore the role of islet-derived sEVs in the development of β cell compensation. To avoid excessive compensation in mice, we selected HFD-fed 6-week male C57BL/6J mice as recipients, and 30 μg of aid-sEVs or nid-sEVs were injected intravenously (twice a week, 4 weeks in total), as shown in Fig. 2A. All recipient mice exhibited similar body weight and fasting glucose after 2 weeks of the last sEV injection (fig. S2, A and B). Aid-sEV treatment led to slightly improved glucose tolerance and insulin sensitivity compared to nid-sEV–treated mice (Fig. 2, B and C). In addition, the counts of Ki67^+^ β cell and β cell mass were higher in aid-sEV–recipient mice than in control animals (Fig. 2, D and E). No changes were detected in terminal deoxynucleotidyl transferase–mediated deoxyuridine triphosphate nick end labeling–positive (TUNEL^+^) β cell after sEV administration (fig. S2C). The above data indicate that aid-sEVs promoted islet β cell adaption.

Given the compensatory effect of aid-sEVs on β cell in vivo, we next ask whether the sEVs can promote β cell proliferation in vitro. Unlike the in vivo results, under aid-sEV stimulation, there was no notable increase in the number of MIN6 cells compared to nid-sEV treatment (fig. S2D). The concentration of insulin in the supernatant did not change with the increase in aid-sEVs (fig. S2E). Consistent with this, aid-sEV treatment did not enhance the sensitivity of MIN6 cells to glucose either (fig. S2F). We then isolated primary pancreatic islets and attempted to investigate whether the sEVs would cause proliferation of β cells in islets. Exposure of isolated murine pancreatic islets to aid-sEVs increased insulin secretion when exposed to high glucose but not low glucose (Fig. 2F). Aid-sEVs also promote the proliferation of β cells in the islets (Fig. 2G) but do not affect the number of TUNEL^+^ β cells (fig. S2G). Collectively, these data indicate that the mode of action of sEVs is paracrine rather than autocrine. We hypothesize that some kinds of neighboring cells of β cells in the islet receive the sEVs and regulate β cell compensation.

### Aid-sEV uptake in recipient cells is partially dependent on F11 Receptor

**Fig. 3.**
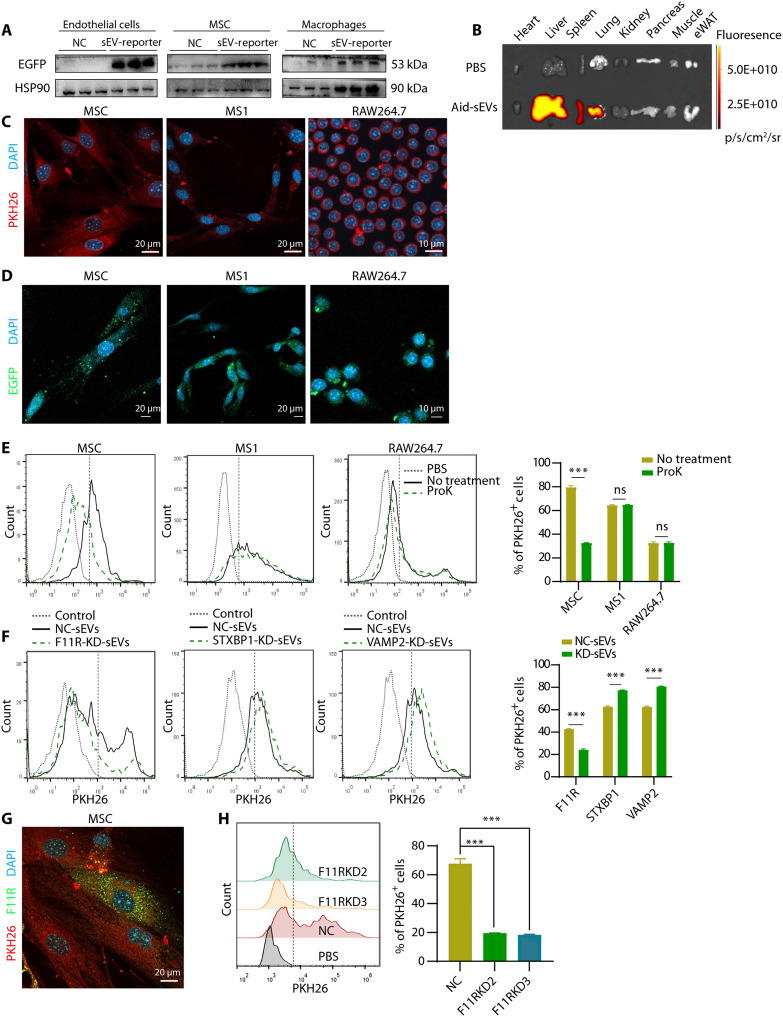
Aid-sEV uptake in recipient cells is partially dependent on F11R. (**A**) MSCs, macrophages, and endothelial cells were isolated by cell sorting after 4 weeks of Ins2-CD63-EGFP-AAV injection. Western blot was performed to detect EGFP expression in these cells (*n* = 3 per group). (**B**) IVIS image of organs from C57BL/6J mice administered with DiR-labeled sEVs intravenously. (**C**) After adding PKH26-labeled sEVs to the medium of MSCs, MS1 cells, and RAW264.7 cells for 24 hours, confocal microscope was used to observe the internalization of PKH26-labeled sEVs by these cells. For MSCs and MS1 cells, magnification, ×20; scale bars, 20 μm. For RAW264.7, magnification, ×40; scale bar, 10 μm. (**D**) Islets were isolated from Ins2-CD63-EGFP-AAV–injected mice and cocultured with MSCs, MS1 cells, and RAW264.7 cells for 24 hours, respectively. Confocal microscope was used to observe the internalization of EGFP^+^ sEVs by these cells. For MSCs and MS1 cells, magnification, ×20; scale bars, 20 μm. For RAW264.7, magnification, ×40; scale bar, 10 μm. (**E**) MSCs, MS1 cells, and RAW264.7 cells were preincubated with 10 μM proteinase K and then treated with PKH26-labeled sEVs. Flow cytometry was used to detect the internalization of PKH26-labeled sEVs by these cells. Cells incubated with phosphate-buffered saline (PBS) alone were used as control (*n* = 3 per group). (**F**) Flow cytometry was conducted to detect the internalization of PKH26-labeled F11R-KD-sEVs, STXBP1-KD-sEVs, and VAMP2-KD-sEVs by MSC. Cells incubated with PBS alone were used as control (*n* = 3 per group). (**G**) Colocalization of F11R (green) and PKH26-labeled sEVs (red) in MSCs. Magnification, ×20; scale bar, 20 μm. (**H**) Flow cytometry analysis of internalization of PKH26-labeled sEVs by F11RKD MSCs. Cells incubated with PBS alone were used as control (*n* = 3 per group). Data are presented as means ± SD. *P < 0.05, **P < 0.01, and ***P < 0.001 by Student’s *t* test [(E) and (F)] or one-way ANOVA (H).

To investigate the transport of the sEVs from islets to adjacent cells, we locally injected Ins2-CD63-EGFP-AAV viruses into HFD-induced obese C57BL/6J mice (starting at 8 weeks of HFD, 10^12^ viral particles per mouse) via pancreatic ductal infusion. After 4 weeks of treatment, we isolated the islets and prepared single-cell suspension. Flow cytometry was used to obtain CD73^+^CD90^+^ cells (identified as MSCs), CD31^+^ cells (identified as endothelial cells), and F4/80^+^ cells (identified as macrophages), respectively (fig. S3A). Western blot analysis showed that EGFP was detected in all three types of cells (Fig. 3A). We then examined whether the aid-sEVs could be transported to distal tissue through blood circulation. DiR-labeled aid-sEVs were injected into the C57BL/6J mice via the tail vein, and fluorescent signals were detected at 6 hours after the administration. The liver, lung, and spleen showed high-intensity signals, while other organs showed the fluorescent was mild (Fig. 3B). We proceeded to explore whether DiR-labeled aid-sEVs were engulfed by MSCs, macrophages, and endothelial cells in other organs. As determined by flow cytometric analyses, the abovementioned cells in different organs can all engulf DiR-labeled aid-sEVs (fig. S3, B and C).

We next conducted an in vitro experiment using PKH26-labeled aid-sEVs and fluorescence analysis to verify the phagocytosis of sEVs by relevant cells. The results showed that the aid-sEVs were internalized into the cytoplasm of the MSCs, macrophages, and endothelial cells (Fig. 3C). Furthermore, we isolated pancreatic islets from mice injected with Ins2-CD63-EGFP-AAV viruses for 4 weeks, which released fluorescently labeled sEVs. After 24 hours of coculture, numerous EGFP^+^ particles were observed within the MSCs, MS1 cells, and RAW264.7 cells (Fig. 3D). Together, these results indicate that the aid-sEVs can be taken up by multiple types of cells in vitro and in vivo.

To elucidate that recipient cells uptake aid-sEVs by directly fusing with the plasma membrane or interacting with extracellular receptors, we used cytochalasin D to inhibit endocytosis and proteinase K to inhibit protein interactions. When cells were treated with cytochalasin D, a decrease in sEV uptake was observed in MS1 and RAW264.7 cells but mild in MSCs (fig. S3D). “Shaving” cell surface proteins through mild proteinase K, the uptake of aid-sEVs by MSCs was weakened (Fig. 3E). However, proteinase K did not reduce the uptake of aid-sEVs by MS1 and RAW264.7 cells (Fig. 3E). In comparison, partial proteolytic removal of sEV surface proteins using mild proteinase K did not affect vesicle morphology or size (fig. S3E) but resulted in a further reduction in aid-sEV uptake by MSCs (fig. S3F). This indicates that the uptake of sEVs by macrophages or endothelial cells is mainly through endocytosis, while MSCs’ aid-sEV uptake depends on protein-protein interactions.

To investigate the surface proteins mediating aid-sEV transport, we performed mass spectrometry analysis on aid-sEVs. A total of 4637 proteins were identified as potent sEV proteins from this analysis (supplied as data S1), of which 34 are membrane proteins (table S3). Among these candidate proteins, only STXBP1, VAMP2, and F11R have been reported to mediate sEV transport (*21*–*23*). To identify the specific molecule that mediates sEV uptake in MSCs, we constructed the F11R–knockdown (KD), STXBP1-KD, and VAMP2-KD MIN6 cell lines and isolated their sEVs (named as F11R-KD-sEVs, STXBP1-KD-sEVs, and VAMP2-KD-sEVs). Western blot was conducted to verify the KD efficiency of F11R, STXBP1, and VAMP2 in these sEVs (fig. S3G). Equal amounts of sEVs were added to the MSC medium. As illustrated in Fig. 3F, compared with negative control (NC)–sEVs, only F11R-KD-sEVs were taken up in a lesser amount by MSCs. F11R can mediate sEV transport by forming dimers (*24*). As expected, fluorescently labeled aid-sEVs and F11R were colocalized in MSCs (Fig. 3G). We next examined the role of F11R in the uptake of aid-sEVs using F11R-KD MSCs (fig. S3H). As illustrated in Fig. 3H, F11R-KD MSCs took up much fewer fluorescently labeled aid-sEVs than controls. These data suggest that F11R is involved in MSCs’ uptake of the aid-sEVs.

### MSCs that received the aid-sEVs are effector cells to promote β cell compensation

**Fig. 4.**
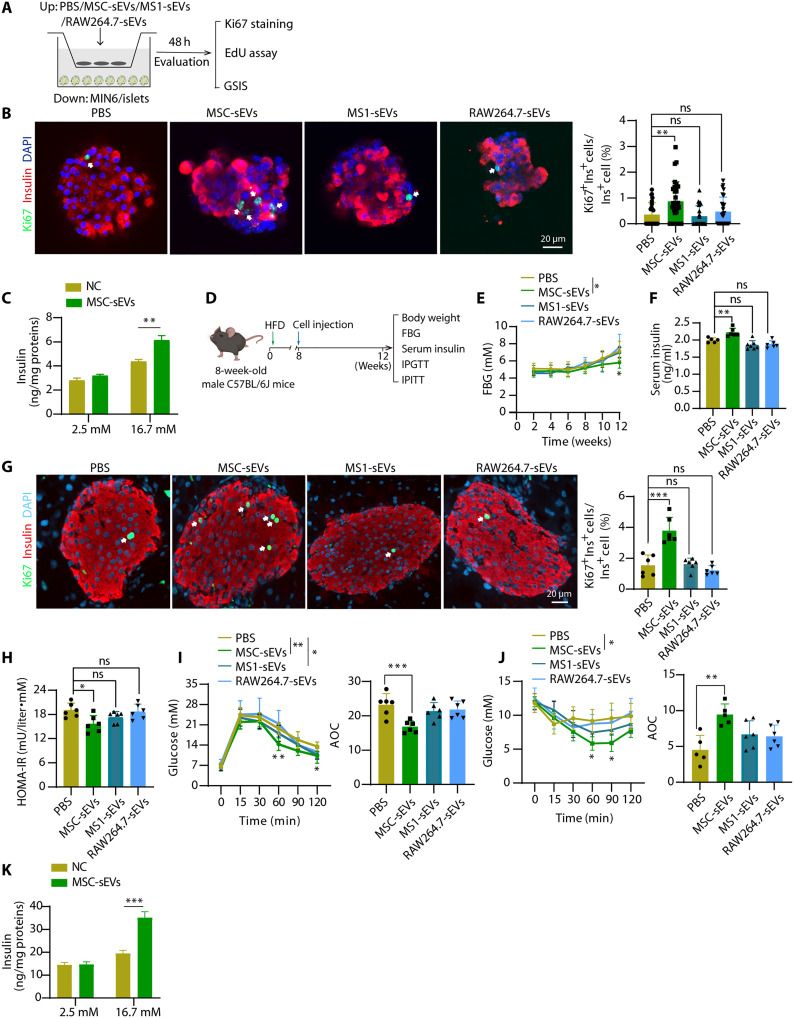
MSCs received the aid-sEVs are effector cells to promote β cell compensation. (**A**) Diagram of coculture system. The icon or graphical images were created in BioRender. Guo, X. (2026) https://BioRender.com/bw6f83d. h, hours.(**B**) Representative images and statistical analysis of Ki67^+^ β cells in islets treated with PBS, MSC-sEVs, MS1-sEVs, and RAW264.7 sEVs. At least 20 islets were analyzed in each group. Magnification, ×20; scale bar, 20 μm. (**C**) GSIS of islets treated with MSC-sEVs (*n* = 3 per group). (**D**) The flowchart of the in vivo experiment designed for detecting the function of different cells on β cell. The icon or graphical images were created in BioRender. Guo, X. (2026) https://BioRender.com/bw6f83d. Eight-week-old C57BL/6J mice were fed with an HFD diet for 8 weeks and administered with PBS, MSC-sEVs, MS1-sEVs, and RAW264.7-sEVs. (**E** and **F**) Fasting blood glucose (FBG) (E) and serum insulin level (F) were measured after 4 weeks of injection (*n* ≥ 5 mice per group). (**G**) Representative images and statistical analysis of Ki67^+^ β cells in PBS-, MSC-sEV–, MS1-sEV–, and RAW264.7-sEV–injected mice (*n* = 6 mice per group). Magnification, ×20; scale bar, 20 μm. (**H** to **J**) Homeostatic model assessment of insulin resistance (HOMA-IR) (H), IPGTT (I), and IPITT (J) were measured after 4 weeks of injection (*n* = 6 mice per group). (**K**) GSIS of islets isolated from MSC-sEV–injected mice (*n* = 3 per group). Data are presented as means ± SD. *P < 0.05, **P < 0.01, and ***P < 0.001 by Student’s *t* test [(C) and (K)] one-way ANOVA [(B) and (F) to (H)] or two-way ANOVA [(E), (I), and (J)].

Given that multiple types of cells can uptake aid-sEVs, we will now explore which cells received the aid-sEVs to promote compensatory β cell function in obesity. We first equipped MSCs, MS1 cells, and RAW264.7 cells with 30 μg of aid-sEVs, respectively (referred to as MSC-sEVs, MS1-sEVs, and RAW264.7-sEVs), and then the transwell coculture strategy was used to find effector cells that stimulate β cell compensation (Fig. 4A). MSC-sEVs can promote the proliferation of primary islet β cells and MIN6 cells, but MS1 and RAW264.7 cells that received the aid-sEVs did not show the corresponding activity (Fig. 4B and fig. S4A). In addition, we found a notable enhancement in GSIS in the primary islets treated by MSC-sEVs, but MS1-sEVs and RAW264.7-sEVs were unable to promote glucose-stimulated insulin secretion (Fig. 4C and fig. S4, B and C). This indicates that MSCs loaded with aid-sEVs have a promoting effect on β cell compensation in vitro.

On the basis of these findings, we investigated whether MSCs loaded with aid-sEVs can improve β cell compensation in vivo. After feeding HFD for 8 weeks, mice were divided into four groups: NC-, MSC-sEV–, MS1-sEV–, and RAW264.7-sEV–treated group (Fig. 4D). There was no notable difference in weight and food intake among the four groups of mice during the study period (fig. S4, D and E). As expected, MSC-sEVs resulted in a decrease in serum blood glucose and an increase in insulin content (Fig. 4, E and F). Compared with other treatment groups, MSC-sEVs promote the proliferation of islet β cells (Fig. 4G). In addition, MSC-sEVs decreased the homeostatic model assessment of insulin resistance (HOMA-IR) values (Fig. 4H). Consistent with this, glucose tolerance and insulin sensitivity improved upon MSC-sEV treatment (Fig. 4, I and J). Last, we isolated the pancreatic islets of all treatment groups of mice. The GSIS results showed that the insulin release was enhanced in MSC-sEV treatment groups but not in MS1-sEV and RAW264.7-sEV groups (Fig. 4K and fig. S4, F and G). These results indicate that MSCs receiving aid-sEVs can promote β cell compensation in vivo.

**Fig. 5.**
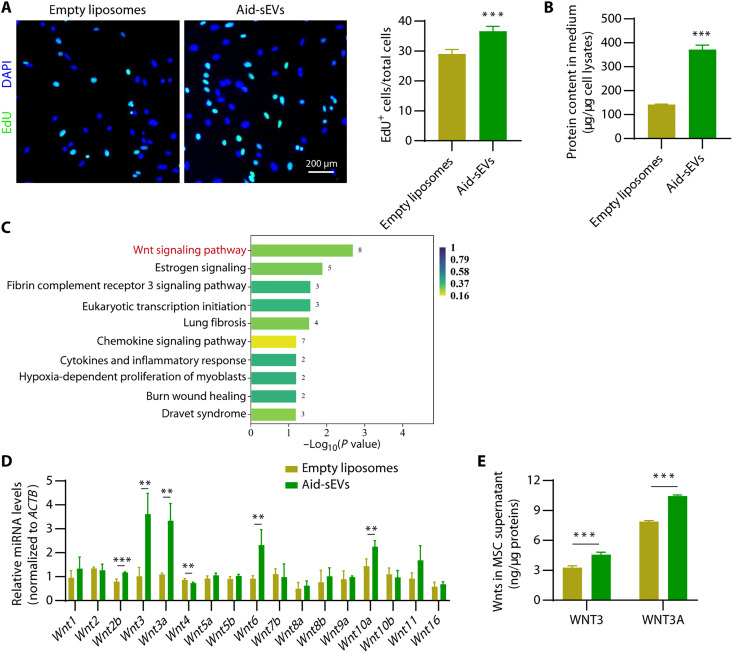
MSCs that received the aid-sEVs are effector cells to promote β cell compensation. (**A**) EdU assay was performed to detect the proliferation of MSCs treated with aid-sEVs (*n* = 3 per group). Magnification, ×10; scale bar, 200 μm. (**B**) Protein content in medium of aid-sEV–treated MSCs. Normalized by cell lysates of donor cells (*n* = 3 per group). (**C**) WikiPathway enrichment analysis of MSC secretome proteins. Proteomic profiling of supernatant from MSCs treated with either aid-sEVs or empty liposomes for 48 hours was performed via mass spectrometry. (**D**) qRT-PCR was performed to detect the expression levels of *Wnt* family in MSCs treated with aid-sEVs. MSCs were subjected to treatment with empty liposomes or aid-sEVs, followed by the isolation of RNA and reverse transcription to cDNA. Subsequently, qRT-PCR was conducted to detect the mRNA levels of *Wnts* in MSCs. *ACTB* was used as normalize mRNAs (*n* = 3 per group). (**E**) The levels of WNT3 and WNT3A in supernatant of MSCs treated with aid-sEVs. MSCs were treated with empty liposomes or aid-sEVs, and then the supernatant and cells were collected. The supernatant was subjected to ELISA detection using a commercial ELISA kit. The cells were subjected to protein isolation and BCA quantification. The levels of WNT3/3A measured by ELISA were normalized to cell protein content to avoid potential variations caused by differences in cell numbers (*n* = 3 per group). Data are presented as means ± SD. *P < 0.05, **P < 0.01, and ***P < 0.001 by Student’s *t* test [(A), (B), (D), and (E)]*.*

Given the compensatory effect of MSC-sEVs on pancreatic islets, we analyzed the impact of aid-sEVs on MSCs. Compared with empty liposomes, aid-sEVs can promote the proliferation of MSCs (Fig. 5A and fig. S4H). In addition, the protein content in the culture supernatant of MSCs stimulated by the aid-sEVs increased (Fig. 5B), indicating that the aid-sEVs have a promoting effect on MSC secretion. The mass spectrometry detection results showed that compared with the empty liposomes, the secretion of some pathway-related proteins, especially the Wnt pathway, increased in the supernatant of the aid-sEV–treated MSCs (Fig. 5C). The results of quantitative polymerase chain reaction (PCR) indicate that the aid-sEVs can enhance the transcription of some Wnts in MSCs (Fig. 5D). Enzyme-linked immunosorbent assay (ELISA) detection further confirmed that the content of some Wnts increased in the supernatant of MSCs stimulated by the aid-sEVs (Fig. 5E). To validation the necessity of MSC uptake of aid-sEVs in the compensatory effect, we applied a coculture system. Conditioned medium was collected from NCD-islets, HFD-islets, and HFD-islets treated with GW4869 (an inhibitor for sEV secretion). MSCs were cultured with different conditioned media for 24 hours and cocultured with MIN6 cells (fig. S4I). The results showed that when the sEV release was inhibited by GW4869, proliferation of MIN6 was reduced (fig. S4J).

### *miR-151* is responsible for the aid-sEV effects on β cell adaption

**Fig. 6.**
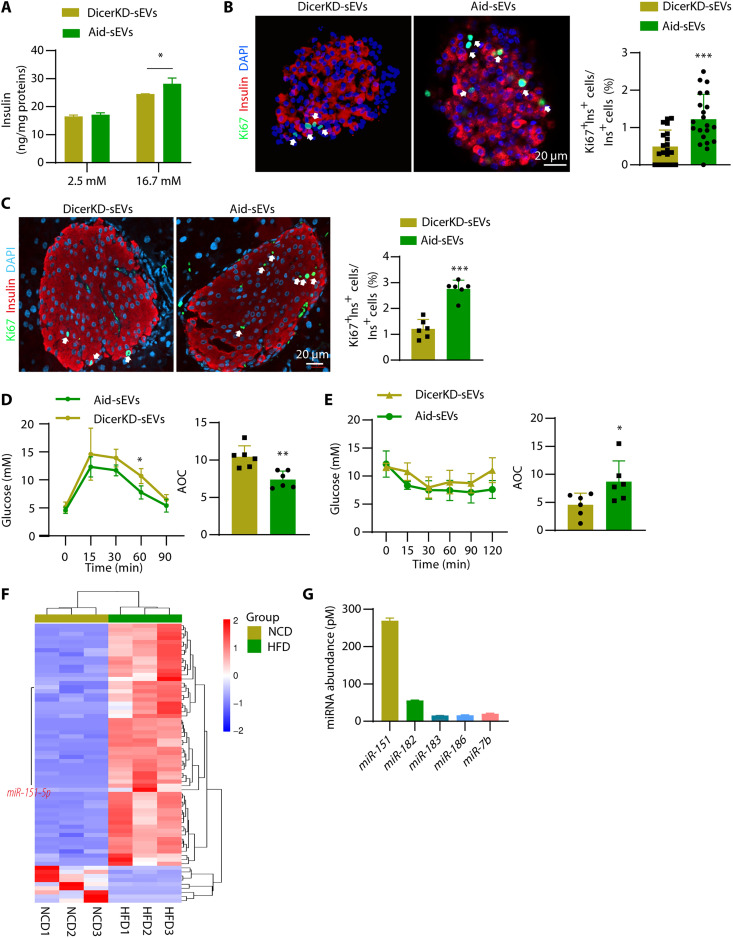
*miR-151* is responsible for the aid-sEV effects on β cell adaption. (**A**) GSIS of islets treated with DicerKD-sEVs and aid-sEVs (*n* = 3 per group). (**B**) Representative images and statistical analysis of Ki67^+^ β cells in islets treated with DicerKD-sEVs and aid-sEVs. At least 20 islets were analyzed in each group. Magnification, ×20; scale bar, 20 μm. (**C**) Representative images and statistical analysis of Ki67^+^ β cells in mice administered with DicerKD-sEVs and aid-sEVs (*n* = 6 mice per group). Magnification, ×20; scale bar, 20 μm. (**D** and **E**) IPGTT (D) and IPITT (E) were measured in mice administered with DicerKD-sEVs and aid-sEVs (*n* = 6 mice per group). (**F**) Heatmap diagram illustrating the differential expression of miRNAs in nid-sEVs and aid-sEVs (*n* = 3 per group). (**G**) The absolute quantification of the top 5 most abundant differentially expressed miRNAs in the miRNA sequencing results (*n* = 3 per group). Data are presented as means ± SD. *P < 0.05, **P < 0.01, and ***P < 0.001 by Student’s *t* test [(A) to (C)] or two-way ANOVA [(D) and (E)].

sEVs carry various bioactive molecules such as miRNAs, proteins, and metabolites (*25*). To determine whether miRNAs in aid-sEVs are required for the observed β cell–adaptive responses, we produced *Dicer*-deficient MIN6 cells (DicerKD-MIN6) to impair miRNA biogenesis (fig. S5A). sEVs from DicerKD-MIN6 cells lacked most miRNAs, confirmed by the substantially lower levels of *miR-375*, common in β cells, in both the cells and their derived sEVs (fig. S5, B and C). Functional evaluation revealed that treatment of primary pancreatic islets with aid-sEVs, but not miRNA-depleted DicerKD-derived sEVs (DicerKD-sEVs), enhanced GSIS and β cell proliferation (Fig. 6, A and B). The role of miRNAs to aid-sEV–mediated effects was further observed in vivo using HFD-fed C57BL/6J mice starting at 6 weeks of diet exposure. After 4 weeks of treatment, mice receiving aid-sEVs showed notable increases in β cell proliferation, insulin sensitivity, and glucose tolerance, whereas administration of DicerKD-sEVs failed to induce similar metabolic improvements (Fig. 6, C to E). These results indicate that miRNAs are the main functional cargo responsible for the ability of aid-sEVs to promote β cell compensation under obese conditions.

**Fig. 7.**
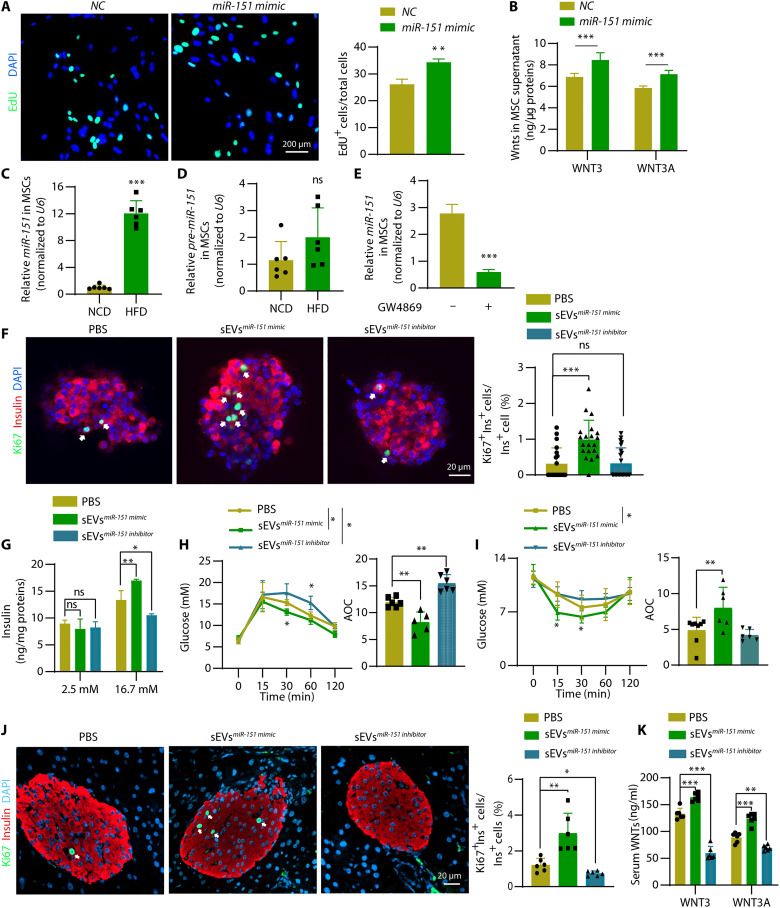
*miR-151* is responsible for the aid-sEV effects on β cell adaption. (**A**) EdU assay was performed to detect the proliferation of MSCs transfected with *NC* and *miR-151 mimic* (*n* = 3 per group). Magnification, ×10; scale bar, 200 μm. (**B**) The levels of WNT3 and WNT3A in supernatant of MSCs transfected with *NC* and *miR-151 mimic* (*n* = 3 per group). (**C** and **D**) qRT-PCR was performed to detect the expression levels of *miR-151* (C) and *pre-miR-151* (D) in MSCs derived from islets of NCD and HFD mice (*n* = 6 mice per group). *U6* was used to normalize miRNA. (**E**) Primary islets were pretreated with GW4869 (20 μM) and then cocultured with MSCs. qRT-PCR was performed to detect the expression level of *miR-151* in MSCs. *U6* was used to normalize miRNA (*n* = 3 per group). (**F**) Representative images and statistical analysis of Ki67^+^ β cells in islets treated with PBS, sEVs^*miR-151* mimic^, and sEVs^*miR-151* inhibitor^. At least 20 islets were analyzed in each group. Magnification, ×20; scale bar, 20 μm. (**G**) GSIS of islets treated with PBS, sEVs^*miR-151* mimic^, and sEVs^*miR-151* inhibitor^ (*n* = 3 per group). (**H** to **K**) Eight-week-old C57BL/6J mice were fed with an HFD diet for 6 weeks and administered with PBS, sEVs^*miR-151* mimic^, and sEVs^*miR-151* inhibitor^ for 4 weeks by tail vein injection (30 μg per mice, twice a week). IPGTT (H), IPITT (I), Ki67^+^ β cells in islets (J), and serum WNT3/3A levels (K) were measured after administration (*n* ≥ 5 mice per group). Data are presented as means ± SD. *P < 0.05, **P < 0.01, and ***P < 0.001 by Student’s *t* test [(A) to (E)], one-way ANOVA [(F), (G), (J), and (K)], or two-way ANOVA [(H) and (I)]*.*

Given the critical role of miRNAs within aid-sEVs, we profiled the miRNAs in nid-sEVs and aid-sEVs to identify candidate miRNAs that mediate β cell compensation. A wide range of changes in the miRNA expression was observed between nid-sEVs and aid-sEVs (Fig. 6F). Among the markedly changed miRNAs, 61 miRNAs were up-regulated, and 15 miRNAs were down-regulated in aid-sEVs compared with nid-sEVs (fig. S5D). Among the markedly up-regulated miRNAs, the top 5 most abundant miRNAs were evaluated (fig. S5E). Further confirmed by quantitative reverse transcription PCR (qRT-PCR) analyses, we found that *miR-151* had the highest abundance in aid-sEVs (Fig. 6G). Consistent with this, the level of *miR-151* also increased in the islets of HFD-fed mice (fig. S5F), and the phenomenon can be recapitulated by treating MIN6 cells with PA (fig. S5G). Meanwhile, *miR-151* has a strong ability to promote MSC proliferation and WNT secretion (Fig. 7, A and B). Transfection with an *miR-151* inhibitor blocked the proliferation and secretion effects of *miR-151* in MSCs (fig. S5, H and I). Although the level of *miR-151* increased in MSCs from HFD-fed mice islets, the level of *pre-miR-151* did not change (Fig. 7, C and D). PA stimulation cannot alter the levels of *miR-151* and *pre-miR-151* in MSCs either (fig. S5, J and K). Using GW4869 to stimulate primary pancreatic islets (inhibit the secretion of sEVs), after coculturing with MSCs, *miR-151* was no longer increased in MSCs (Fig. 7E). All the above results indicate that the elevated *miR-151* in MSCs is exogenous and it may be responsible for the aid-sEV effects on β cell compensation.

Given that the increased of *miR-151* in compensatory pancreatic islets, we first investigated the effect of overexpression of *miR-151* on MIN6 cells. Ectopic expression of *miR-151* cannot alter the proliferation and insulin secretion of MIN6 cells (fig. S5, L and M). This indicates that the compensatory promoting effect of *miR-151* on β cells is likely to be achieved through the form of sEVs. To explore this idea, we prepared *miR-151* overexpression and KD sEVs (referred to as sEVs^*miR-151* mimic^ and sEVs^*miR-151* inhibitor^) by transfecting DicerKD-MIN6 cells with *miR-151* mimic or loading aid-sEVs with *miR-151* inhibitor, respectively. To test whether *miR-151* exerts β cell compensation effects in vitro, we used the sEVs^*miR-151* mimic^ and sEVs^*miR-151* inhibitor^ to directly treat primary pancreatic islets. We found that the sEVs^*miR-151* mimic^, but not the sEVs^*miR-151* inhibitor^, promoted the proliferation of β cells and insulin secretion (Fig. 7, F and G). However, sEVs^*miR-151* mimic^ and sEVs*^miR-151 inhibitor^* cannot alter the proliferation and insulin secretion of MIN6 cells (fig. S5, N and O). This suggests that sEVs^*miR-151* mimic^ promotes β cell compensation using certain types of cells within the islet. For in vivo studies, sEVs^*miR-151* mimic^ or sEVs^*miR-151* inhibitor^ was intravenously injected into HFD-fed mice (starting at 6 weeks of HFD), with phosphate-buffered saline (PBS) as the NC (fig. S5P). The *miR-151* mimic and *miR-151* inhibitor are efficiently delivered into key metabolic tissues as shown by qRT-PCR (fig. S5Q). After 4 weeks of treatment with sEVs^*miR-151* mimic^ (30 μg per mouse, twice per week), mice exhibited improved glucose and insulin tolerance compared to control (Fig. 7, H and I). In addition, in vivo delivery of the sEVs^*miR-151* mimic^ led to higher levels of Ki67^+^ β cell (Fig. 7J). As expected, ELISA analysis showed that some WNTs were increased in serum after sEV^*miR-151* mimic^ treatment (Fig. 7K). Overall, these results demonstrate that *miR-151* contributes to the beneficial effects of aid-sEVs on β cell compensation both in vivo and in vitro.

**Fig. 8.**
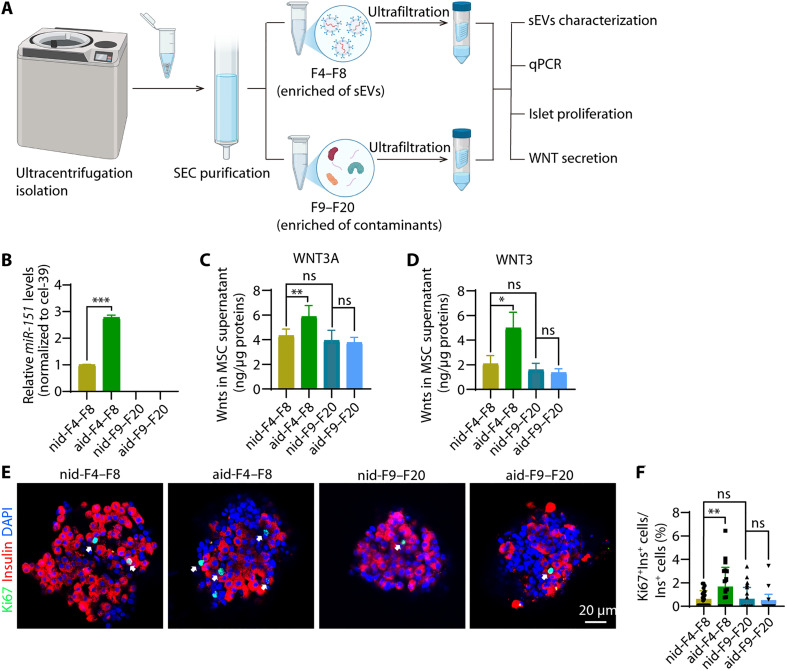
Purified aid-sEVs promote islet compensation. (**A**) Schematic diagram of sEV purification. Nid-sEVs and aid-sEVs were isolated by ultracentrifugation and subjected to a SEC column to purification. Fractions 4 to 20 were collected in which fractions 4 to 8 (F4–F8) was enriched of sEVs and fractions 9 to 20 (F9–F20) was enriched of soluble contaminants. F4–F8 and F9–F20 were concentrated using ultrafiltration and allocated to functional assays. The icon or graphical images were created in BioRender. Guo, X. (2026) https://BioRender.com/m618tu3. (**B**) qRT-PCR was performed to detect the expression level of *miR-151* in F4–F8 and F9–F20 of different groups (*n* = 3 per group). (**C** and **D**) The levels of WNT3 and WNT3A in supernatant of MSCs treated with F4–F8 and F9–F20 in different groups. MSCs were treated with nid-F4–F8, nid-F9–F20, aid-F4–F8, and aid-F9–F20, and then the supernatant and cells were collected. The supernatant was subjected to ELISA detection using a commercial ELISA kit. The cells were subjected to protein isolation and BCA quantification. The levels of WNT3/3A measured by ELISA were normalized to cell protein content to avoid potential variations caused by differences in cell numbers (*n* = 3 per group). (**E** and **F**) Primary islets were incubated with nid-F4–F8, nid-F9–F20, aid-F4–F8, and aid-F9–F20. Ki67 staining were performed to detect the proliferation of islets. For Ki67 staining, at least 20 islets were analyzed in each group. Magnification, ×20; scale bar, 20 μm. Data are presented as means ± SD. *P < 0.05, **P < 0.01, and ***P < 0.001 by Student’s *t* test and one-way ANOVA [(B) to (F)].

On the basis of the central role of sEVs*^miR-151^* in β cell compensation. We purified sEVs by size exclusion chromatography (SEC) to exclude the impact of soluble contaminants (Fig. 8A). As characterized by TEM, NTA, and Western blot, fractions 4 to 8 were enriched of purified sEVs (fig. S6, A to D). We also collected fractions 9 to 20 to concentrate soluble contaminants and found that *miR-151* was undetectable in fractions 9 to 20 (Fig. 8B). Furthermore, purified aid-sEVs, rather than soluble contaminants, promoted WNT3, WNT3A secretion (Fig. 8, C and D), and β cell proliferation (Fig. 8, E and F).

### *miR-151* energizes MSCs by targeting Krüppel-like factor 9

**Fig. 9.**
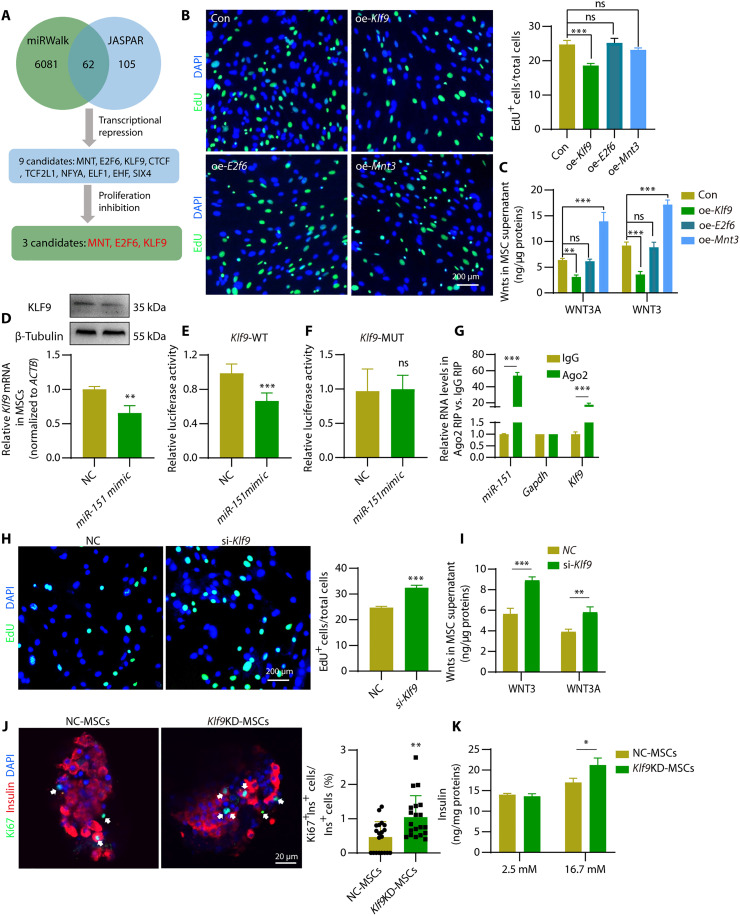
*miR-151* energizes MSCs by targeting KLF9. (**A**) miRWalk and JASPAR websites were used to predict the target genes of *miR-151*. (**B**) EdU assay was performed to detect the proliferation of MSC oe-*Klf9*, oe-*E2f6*, or oe-*Mnt3* (*n* = 3 per group). (**C**) WNT3 and WNT3A levels in supernatant of MSC oe-*Klf9*, oe-*E2f6*, or oe-*Mnt3* (*n* = 3 per group). (**D**) Expression of *Klf9* was measured by qRT-PCR and Western blot in MSCs transfected with *miR-151 mimic* (*n* = 3). (**E** and **F**) Relative luciferase activity of human embryonic kidney (HEK) 293T cotransfected with *miR-151 mimic* and a luciferase reporter containing *Klf9*-WT (E) or *Klf9*-MUT (F). The data are presented as the relative ratio of *Renilla* luciferase activity to firefly luciferase activity (*n* = 3 per group). (**G**) Anti-Ago2 RNA immunoprecipitation was performed in MSCs transiently overexpressing *miR-151*, followed by qRT-PCR to detect *Klf9* associated with Ago2 [nonspecific immunoglobulin G (IgG) served as NC] (*n* = 3 per group). (**H**) EdU assay was performed to detect the proliferation of MSCs transfected with *Klf9* siRNA (*n* = 3 per group). (**I**) WNT3 and WNT3A levels in supernatant of MSCs transfected with *Klf9* siRNA (*n* = 3 per group). (**J** and **K**) Primary islets were incubated with supernatant collected from *Klf9* siRNA–transfected MSCs. Ki67 staining (J) and GSIS (K) were performed to detect the proliferation and GSIS of islets. For Ki67 staining (J), at least 20 islets were analyzed in each group. Magnification, ×20; scale bar, 20 μm. Data are presented as means ± SD. *P < 0.05, **P < 0.01, and ***P < 0.001 by Student’s *t* test [(D) to (K)] or one-way ANOVA [(B) and (C)].

On the basis of the role of *miR-151* in stimulating MSC proliferation and WNT protein secretion, in silico analyses were performed to identify downstream targets. Target prediction was conducted using the miRWalk and JASPAR databases, and the workflow of prediction is summarized in Fig. 8A. Intersection analysis identified 62 candidate genes predicted to be both *miR-151* targets and transcriptional regulators of WNTs. The study further predicted that, on the basis of transcriptional suppression and relevance to proliferative regulation, *Klf9*, *E2f6*, and *Mnt3* are the most likely functional targets of *miR-151* (Fig. 9A). Given that the target gene of *miR-151* will inhibit the secretion of WNTs in MSCs, we used ectopic expression strategy to investigate the effects of *Klf9, E2f6*, and *Mnt3* on MSCs. 5-Ethynyl-2′-deoxyuridine (EdU) and ELISA assays showed that MSC overexpression of *Klf9* (oe-*Klf9*) exhibited a notable inhibition of proliferation and WNT secretion phenotype compared with *E2f6* and *Mnt3* (Fig. 9, B and C). In addition, *Klf9* expression was reduced in compensatory islets (fig. S7A). Consistent with this, the expression of *Klf9* is also reduced in the pancreatic islet of HFD-fed recipients treated with aid-sEVs (fig. S7B). Furthermore, we found that the MSCs expressed much less KLF9 after transfection with the *miR-151* mimic (Fig. 9D). All the above observations are sufficient to encourage us to explore the targeted relationship between *miR-151* and KLF9.

Sequence analysis identified a conserved *miR-151* recognition site within the 3′ untranslated region (3′UTR) of *Klf9*, which is conserved across human and mice (fig. S7C). To validate this interaction, we generated luciferase reporter constructs containing either the wild-type *Klf9* 3′UTR (2190 nt; *Klf9*-WT) or a mutated *miR-151* binding site (*Klf9*-MUT). Reporter assays showed that a *miR-151* mimic reduced luciferase activity driven by the *Klf9*-WT construct (Fig. 9E). In comparison, no change was observed with the *Klf9*-MUT reporter (Fig. 9F). RNA immunoprecipitation using an Ago2 antibody revealed substantial enrichment of *Klf9* mRNA within the RNA-induced silencing complex (Fig. 9G and fig. S7D). We next evaluated the importance of *miR-151*–mediated *Klf9* suppression on MSCs and β cell compensation. As shown in fig. S7E, the KD efficiency of *si-Klf9* in MSCs reached 60%. After transfection with *siRNA-Klf9* for 24 hours, MSCs exhibited increased proliferation and secretion of WNTs (Fig. 9, H and I). In addition, using the cocultivation strategy, *Klf9KD-*MSCs (si-*Klf9* KD in MSCs) led to an increase in the proliferation of pancreatic islet β cells and elevated insulin secretion (Fig. 9, J and K). However, treatment with both si-*Klf9* and aid-sEVs did not further improve the compensatory effect, compared to the cells treated with either si-*Klf9* or aid-sEVs alone (fig. S7, F and G). These data demonstrate that the *miR-151–Klf9* axis plays a role in modulating β cell compensation.

To explain the molecular mechanism by which KLF9 promotes MSC proliferation and WNT secretion, we will now attempt to identify downstream partners of KLF9. With the help of the JASPAR website, we predicted that KLF9 can bind to various proliferation-related transcription factor promoter regions, as well as other WNT promoter regions besides WNT2 (fig. S7, H and I). We focused on measuring the binding of KLF9 to the *Ccnd1* and *Wnt3a* promoters. The dual luciferase reporter assay confirmed that KLF9 can bind to the promoter regions of *Ccnd1* and *Wnt3a* (fig. S7, J and K). As expected, ectopic expression of *Klf9* in MSCs resulted in a decrease in the expression levels of *Ccnd1* and *Wnt3a* (fig. S7, L and M). This indicates the inhibitory effect of KLF9 on the expression of *Ccnd1* and *Wnt3a*. The inhibitory effect of KLF9 on *Ccnd1* confirms its proliferative inhibitory effect, while its regulatory effect on *Wnt3a* indicates its compensatory effect on β cells.

## DISCUSSION

**Fig. 10.**
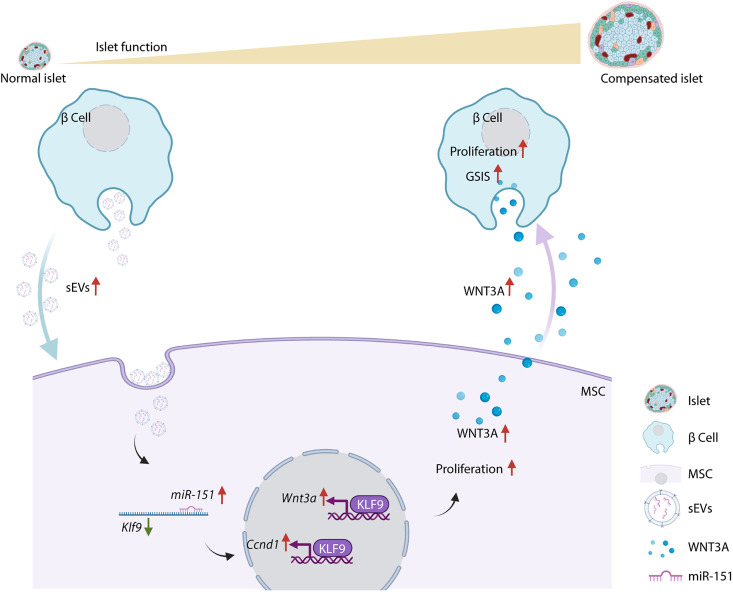
A working model that illustrates the mechanism by which sEV *miR-151* promotes islet compensation. During the compensatory phase, sEV *miR-151* secreted by islets is markedly up-regulated. sEV *miR-151* promotes the proliferation and Wnt secretion of islet MSCs by targeting KLF9. The phenotypic transformation of MSCs further enhances β cell functional compensation. The icon or graphical images were created in BioRender. Guo, X. (2026) https://BioRender.com/nxqaosj.

Insulin resistance is a central pathophysiological abnormality underlying both obesity and T2D, whereas adaptive responses of pancreatic islets are crucially involved in preserving systemic metabolic homeostasis. These results demonstrate that aid-sEVs protect glucose metabolism, as evidenced by improved glucose tolerance and enhanced insulin sensitivity in obese mice. Disruption of the miRNA cargo within these vesicles abrogated their potential to support β cell compensatory responses, indicating that sEV-associated miRNAs are essential mediators of this adaptive process. Among these, *miR-151* was identified as a highly enriched component of aid-sEVs and emerged as a pivotal regulator of islet adaptation. Restoration of *miR-151* expression in miRNA-depleted sEVs reproduced the procompensatory effects of intact aid-sEVs. KLF9 was confirmed as a direct target of *miR-151*, establishing the *miR-151–*KLF9 regulatory axis as a key pathway of β cell compensation in obesity (Fig. 10).

Recent research highlights the crucial role of sEVs in the development and progression of metabolic diseases, observed in both animal models and patients with T2D (*26*, *27*). Using a β cell–specific Ins2-CD63-EGFP-AAV tracing vector, increased circulating sEV levels were observed in HFD-fed mice compared to those on an NCD. These results are consistent with clinical data showing increased sEV concentrations in the blood of diabetic patients relative to metabolically healthy individuals (*28*). The presence of β cell–specific markers, such as insulin and *miR-375*, within circulating sEVs confirms their pancreatic islet origin, thus providing a mechanistic basis for investigating sEV-mediated communication between β cells and surrounding cells during obesity-related metabolic stress.

Obesity not only increases the number but also alters the molecular composition of islet-derived sEVs. Since sEV-associated miRNAs are known to influence metabolic phenotypes (*29*), the special focus of this study was to analyze miRNA cargo. Comparative miRNA profiling revealed a distinct signature in sEVs isolated from HFD-fed mice, with 61 miRNAs being up-regulated and 15 down-regulated compared to NCD-derived sEVs. Moreover, *miR-151* was among the obesity-enriched miRNAs, as higher circulating levels have been previously observed in prediabetic and insulin-resistant individuals (*30*). Consistent with this observation, *miR-151* levels increased in pancreatic islets after exposure to PA and were actively secreted by MIN6 β cells under lipotoxic conditions. Besides this, using GW4869 to inhibit sEV release decreased PA-induced *miR-151* secretion, confirming that *miR-151* export is mediated predominantly through sEV pathways. Further, *miR-151* was the most abundant of the up-regulated miRNAs, a feature considered crucial for functional delivery to recipient cells via sEVs. Functional screening of the five most abundant miRNAs showed that *miR-151* specifically promoted both MSC proliferation and WNT ligand secretion, whereas the remaining candidates had limited or no detectable effects on MSC function (fig. S5, R and S). These results support selecting *miR-151* as a key effector of aid-sEV–mediated β cell compensation. Although the role of other miRNAs cannot be excluded, further investigation is required to fully elucidate the combined or redundant roles of other sEV-associated miRNAs in β cell–adaptive responses. Ultracentrifugation-based sEV isolation may coenrich RNA-protein complexes, including Ago2-associated miRNAs. To address whether *miR-151* is genuinely sEV associated, we pharmacologically inhibited sEV secretion using GW4869 (an inhibitor of ceramide-mediated vesicle release). Treatment of donor islets with GW4869 resulted in a notable decrease in *miR-151* levels in recipient MSCs (Fig. 7E). Furthermore, we purified sEVs by a SEC column and conducted functional experiments with purified sEVs and concentrated soluble contaminants (Fig. 8 and fig. S6). These functional evidence supports that *miR-151* transfer is dependent on sEV secretion, thereby mitigating concerns about free RNA-protein contamination contributing to this specific observation. The results showed that β cells are the main source of sEV-associated *miR-151* under obese conditions. Considering the combined role of these cell types in glucose homeostasis (*31*–*33*), the selective induction of β cell–derived *miR-151* suggests that this miRNA might act as an intrinsic distress signal released during metabolic stress.

Considering the notable role of sEV-associated miRNAs as mediators of intercellular communication (*34*), the study further examined the distribution and functional impact of islet-derived *miR-151*. Coculture experiments showed that aid-sEVs do not directly stimulate β cell proliferation but instead enhance β cell mass within intact islets, indicating an indirect, paracrine mode of action. Selective delivery of *miR-151* to MSCs depended on the junctional adhesion molecule F11R, expressed on both aid-sEVs and recipient MSCs. F11R-KD considerably impaired this sEV uptake by MSCs. These findings are consistent with previous reports describing selective sEV uptake mediated by surface protein interactions (*35*, *36*). Although approximately 34 membrane proteins were identified within the aid-sEV proteome, further studies are required to determine whether other receptors contribute to MSC-specific uptake. The spectrum of recipient cells for aid-sEVs included not only MSCs but also macrophages and endothelial cells. However, uptake of aid-sEVs by macrophages or endothelial cells did not contribute to β cell compensatory responses. This observation suggests that aid-sEVs may exert additional biological functions beyond regulating β cell adaptation and requires further investigation. Moreover, many studies, including the report by López-Bermudo *et al.* (*37*), demonstrate that the hepatokines such as HGF, fibroblast growth factor 21 (FGF21), and ANGPTL8 enhance β cell function in metabolic disorders. In this study, the effects of aid-sEVs on key hepatokines were evaluated. The findings indicate that exposure to aid-sEVs increased FGF21 expression in AML12 hepatocytes, while levels of ANGPTL8, HGF, and SerpinB1 remained unchanged. The specific induction of FGF21 indicates a potential signaling pathway for liver-islet communication (fig. S7L). However, the precise role of islet-derived sEVs in regulating hepatic metabolic pathways in T2D requires comprehensive, system-level research.

Pancreatic β cells show considerable plasticity when responding to the chronic state of insulin resistance (*38*). The progressive failure of these adaptive mechanisms is a key pathogenic event that leads to β cell dysfunction and the eventual development of diabetes (*39*). Therefore, strategies that improve the functional β cell mass by harnessing intrinsic adaptive responses are considered as promising treatments for T2D. Our findings suggest that pancreatic islets respond to insulin-resistant conditions by releasing *miR-151–*enriched sEVs, which are transferred to MSCs and promote β cell–adaptive responses. These results were confirmed through in vivo and in vitro data. Repeated intravenous injections of 30 μg of sEVs per mouse improved insulin sensitivity and increased β cell mass in obese mice. In vitro coculture experiments showed that islet-derived sEV-associated *miR-151* promotes compensatory responses in primary pancreatic β cells.

In summary, adaptive islet–derived sEVs were identified as key regulators of β cell compensation during obesity. Administration of aid-sEVs enhanced β cell mass and improved insulin sensitivity in both in vivo and in vitro models, whereas KD of sEV-associated *miR-151* abolished these protective effects. These findings identify islet-derived sEV-associated *miR-151* as a key endogenous self-rescue signaling mediator during obesity, highlighting its potential as a therapeutic target for the treatment of metabolic disorders.

## MATERIALS AND METHODS

### Cell culture

Both cell types (MIN6 cells and MSCs) were allowed to grow at 37°C in a humidified incubator containing 5% CO_2_. Cells were cultured in Dulbecco’s modified Eagle’s medium supplemented with 15% fetal bovine serum (FBS), penicillin (100 IU/ml), streptomycin (100 μg/ml), and 1% β-mercaptoethanol. For sEV collection, primary islets or cells were cultured under serum-free conditions using sEV-depleted FBS. Commercially prepared sEV-depleted FBS was obtained from Echo Biotech (Beijing, China).

### Isolation of islet MSCs

Primary pancreatic islets were isolated following previously reported procedures (*40*). Isolated islets were transferred to T25 culture flasks and allowed to adhere. MSCs were extended from the adhered islets after ~72 hours. Migrated cells were expanded and phenotypically characterized according to the criteria established by the International Society for Cellular Therapy (*41*), using a panel of surface markers to characterize MSCs via flow cytometry. Specifically, cells were phenotyped using CD73^+^ and CD90^+^ as positive markers, while CD31^−^ and CD45^−^ were used as negative markers to exclude hematopoietic and endothelial lineages. Stained cells were analyzed by a BD FACSCelesta flow cytometer. CD73^+^ CD90^+^ cells were gated within CD31^−^ CD45^−^ population. Isotype-matched antibodies were used to set positive gating thresholds.

### Animal experiments

C57BL/6J mice were purchased from Hangzhou Ziyuan Laboratory Animal Technology Co. Ltd. Animals were housed under specific pathogen–free conditions with a 12-hour light/dark cycle at 23° to 25°C at China Pharmaceutical University. All experimental procedures were reviewed and approved by the Ethics Committee of China Pharmaceutical University (ethics number: 2024-05-015). For sEV administration, mice were fed either an NCD or an HFD (60% fat, 20% carbohydrate, and 20% protein) for 6 weeks. Afterward, sEVs (30 μg per mouse) in 100 μl of PBS were injected intravenously twice weekly. Control animals received the same volume of PBS. Body weight and fasting blood glucose (FBG) levels were measured weekly.

Recombinant RIP (Rat Insulin2 Promoter)–CD63–EGFP-AAV8 and NC viruses were generated by Nanjing Corues Biotechnology. Mice were anesthetized and administered 1 × 10^12^ viral particles via intraductal injection, as previously described (*42*). Briefly, viral preparations were diluted in 100 μl of saline and infused into the pancreatic duct at 6 μl/min over 25 min using an R462 perfusion pump (RWD Life Sciences, Shenzhen, China).

For cell-based treatments, MSCs, MS1 cells, and RAW264.7 cells were incubated with aid-sEVs and then transplanted into the pancreatic subcapsular space at a density of 2.5 × 10^5^ cells per mouse. Control groups received PBS alone. Body weight and FBG were measured weekly.

### Metabolic characterization

For FBG measurement, mice were subjected to a 16-hour fasting with access to water. Blood samples were collected from tail vein, and levels of blood glucose were measured using a blood glucose meter (OMRON i-sens 631, Japan). For glucose tolerance testing (GTT), mice were fasted for 14 to 16 hours with free access to water and then injected intraperitoneally with d-glucose (1 g/kg of body weight). Blood glucose levels were measured from the tail vein at predetermined time points. For insulin tolerance testing (ITT), mice were fasted for 8 hours before intraperitoneal injection of insulin (0.5 U/kg of body weight). The area of curve (AOC) of GTT and ITT were calculated by area under the curve with subtraction of the basal glucose (*43*). All the procedures were approved by the Ethics Committee of China Pharmaceutical University (ethics number: 2024-05-015).

### sEV isolation and characterization

Conditioned culture medium was centrifuged sequentially: 300*g* for 10 min to discard cells, 2000*g* for 10 min to remove dead cells, and 10,000*g* for 30 min to clear cellular debris and large particles. The supernatant was then ultracentrifuged at 120,000*g* for 90 min to pellet sEVs. These pellets were resuspended in PBS, washed, and ultracentrifuged again at 120,000*g* for 90 min. The final sEV preparations were resuspended in a minimal volume of PBS for downstream analysis. Ultracentrifugation was carried out using a Beckman Optima CPN-80 with a type 70 fixed-angle titanium rotor (23° angle, K-factor 44).

For NTA, sEV samples were diluted and analyzed using a NanoSight NS300 instrument (Malvern Panalytical, UK) with a 488-nm blue laser. For TEM, sEVs were fixed in 2.5% glutaraldehyde at 4°C overnight, transferred onto formvar carbon–coated copper grids, and contrast stained with 4% uranyl acetate for 5 min at room temperature. Ultrastructural imaging was performed using a Hitachi HT7700 Exalens transmission electron microscope.

For the quantification of sEVs, BCA assay was performed according to MISEV2018 guidelines (*44*). In addition, the NTA results were normalized to the total cellular protein amount from the corresponding donor cells (*45*).

For sEV purification, the sEVs isolated by ultracentrifugation were suspended by 1 ml of PBS and subjected to SEC according to manufacturer’s instructions (ES914, Echo Biotech, China). Briefly, the column was equilibrated with 20 ml of PBS, after which 1 ml of samples were loaded onto the column. A total of 20 fractions of the eluate (0.5 ml per fraction) were collected, in which fractions 4 to 8 were enriched with sEVs and fractions 9 to 20 contains soluble contaminants. The sEV-enriched fractions (fractions 4 to 8) and contaminant-enriched fractions (fractions 9 to 20) were concentrated by Pall centrifugal filter (molecular weight cutoff = 100 and 1 kDa, separately).

### Flow analysis of sEVs

sEVs were attached to 4-μm aldehyde/sulfate latex beads for flow cytometric analysis. Briefly, 30 μg of sEVs were incubated with 10 μl of latex beads at 37°C for 15 min. Next, 1 ml of PBS was added, and the suspension was gently rotated at 37°C for 2 hours for proper conjugation. The conjugation reaction was terminated by adding 10 μl of 1 M glycine, followed by incubation at room temperature for 30 min. Bead-bound sEVs were permeabilization with Triton X-100, followed by proteinase K to digest both external and internal proteins, or incubation with proteinase K alone to digest proteins externally associated with the vesicles. After treatment, samples were incubated with fluorochrome-conjugated antibodies specific for the markers of interest.

For the detection of EGFP^+^ sEVs, serum sEVs were isolated and attached to 4 μm aldehyde/sulfate latex beads in the same manner and then subjected to flow cytometry analysis.

Data were acquired on a BD FACSCelesta flow cytometer and analyzed with FlowJo v10. Isotype-matched antibodies were used to set positive gating thresholds.

### Human blood samples

Clinical serum samples (*n* = 54) and their related clinicopathological information were obtained from Zhongda Hospital, affiliated with Southeast University (Nanjing, China). The study population included individuals aged 18 to 75 years old. Clinical assessments, including measurements of serum triglycerides and cholesterol, were performed. Detailed patient characteristics are summarized in tables S1 and S2. For serum preparation, all participants fasted overnight, and 5 ml of peripheral venous blood was collected in the morning using a closed blood collection system. Samples were processed immediately for serum separation, and no hemolysis was observed. Serum aliquots were stored at −80°C until sEV isolation.

Serum samples (500 μl) were centrifuged at 10,000*g* for 30 min to remove large particulates, followed by ultracentrifugation at 120,000*g* for 90 min to pellet sEVs. The resulting sEVs were resuspended in 200 μl of PBS for further analyses.

Written informed consent was obtained from all participants, and all procedures were conducted as per the Declaration of Helsinki. Ethical approval was received by the Ethics Committee of Zhongda Hospital, Southeast University (Nanjing, China; approval no. 2018ZDSYLL132-P01).

### sEV treatment

For in vivo administration, sEVs were delivered intravenously at a dose of 30 μg per mouse in 100 μl of PBS, twice weekly. Control animals received the same volume of PBS. NC oligonucleotides and *miR-151* inhibitors were loaded into sEVs using Exo-Fect Exosome Transfection Reagent (SBI, CA, USA) according to the manufacturer’s protocol.

To generate control sEVs (sEVs*^NC^*) and *miR-151–*enriched sEVs (sEVs*^miR-151 mimic^*), NC or *miR-151* mimics were transfected into Dicer-KD MIN6 cells. For in vitro experiments, 10 μg of sEVs were added to culture medium containing 5 × 10^5^ MSCs.

Synthetic *miR-151* mimics (sequence identical to *mmu-miR-151-5p*) and *miR-151* inhibitors (reverse complementary to *mmu-miR-151-5p*) were synthesized by GenePharm (Shanghai, China). Oligonucleotide sequences are provided in table S6.

### sEV uptake in vivo

sEVs were fluorescently labeled with DiR dye (Beyotime, Shanghai, China) following the manufacturer’s protocols and were injected intravenously. After treatment, major organs were collected and imaged using an IVIS Spectrum imaging system (PerkinElmer, USA) to evaluate sEV biodistribution (*46*). To determine cell type–specific uptake of sEVs, liver and lung tissues were enzymatically dissociated with collagenase II. For identification of MSCs, single-cell suspensions were stained with PerCP-Cy5.5–conjugated anti-CD31 (BioLegend, catalog no. 102420), fluorescein isothiocyanate (FITC)–conjugated anti-CD90 (BioLegend, catalog no. 157213), allophycocyanin (APC)-conjugated anti-CD45 (BioLegend, catalog no. 147707), and phycoerythrin (PE)–conjugated anti-CD73 (BioLegend, catalog no. 101703) antibodies. Endothelial cells were identified using PerCP-Cy5.5–conjugated anti-CD31 antibodies, whereas macrophages were detected using PE-conjugated anti-F4/80 antibodies (BioLegend, catalog no. 111603). All samples were analyzed by flow cytometry using a BD FACSCelesta cell analyzer. Data were processed using a sequential gating strategy that excluded digestion-associated debris. MSCs were identified within the CD31^−^CD45^−^ population, whereas macrophages and endothelial cells were gated as F4/80^+^ and CD31^+^ populations, respectively. Flow cytometric data were analyzed using FlowJo v10, with isotype controls used to define the boundaries of positive staining.

### sEV uptake in vitro

sEVs were fluorescently tagged with PKH26 dye (Beyotime, Shanghai, China) based on the manufacturer’s protocol and then incubated with MSCs, MS1 cells, and RAW264.7 macrophages. To investigate uptake mechanisms, MSCs were preincubated with proteinase K (10 μg/ml) or cytochalasin D (5 μg/ml) before exposure to PKH26-labeled sEVs. After incubation for the indicated durations, sEV uptake was examined by flow cytometry or confocal laser scanning microscopy. Flow cytometric data were acquired on a BD FACSCelesta cell analyzer and analyzed with FlowJo software (v10). Untreated cells lacking PKH26-labeled sEVs served as NCs for gating positive fluorescence signals.

### Cell coculture

For sEV loading, 10 μg of aid-sEVs were added to culture medium containing 5 × 10^5^ MSCs, MS1 cells, or RAW264.7 cells, and the mixture was incubated for 24 hours to form sEV-loaded cells (MSC-sEVs, MS1-sEVs, and RAW264.7-sEVs). MIN6 cells, isolated pancreatic islets, and sEV-loaded cells were then cocultured in a Transwell system. MIN6 cells or islets were seeded in the lower chamber, while MSC-sEVs, MS1-sEVs, or RAW264.7-sEVs were allowed to grow in Transwell inserts (Corning; 1.0-μm pore size) positioned above the culture plate.

### Mass spectrometry analysis

Aid-sEVs were isolated by ultracentrifugation and processed for protein extraction, followed by mass spectrometry analysis performed by Applied Protein Technology (Shanghai, China). For proteomic profiling of MSC-conditioned medium, MSCs were treated with aid-sEVs or empty liposomes. Culture supernatants were collected and lyophilized, with 15 ml of medium prepared per sample. The resulting freeze-dried material was used for protein extraction and mass spectrometry analysis by Applied Protein Technology (Shanghai, China).

### ELISA assay

The concentrations of WNT3A, WNT3, and insulin in mouse serum or cell culture supernatants were quantified using commercially available ELISA kits (Jiangsu Boshen Biotechnology Co. Ltd., China) as per the manufacturer’s guidelines. Briefly, 10 μl of each sample was added to the ELISA plate, and protein levels were determined from standard curves generated using known concentrations. The donor cells were also processed for total protein isolation and quantified using a BCA assay. ELISA results were normalized to total cellular protein content to minimize variability arising from differences in cell density.

### Cell proliferation detection

For EdU incorporation analysis, cells were seeded in 24-well plates, and proliferation was evaluated using the BeyoClick EdU Cell Proliferation Kit with Alexa Fluor 488 (Beyotime Institute of Biotechnology), following the manufacturer’s recommendations. For Ki67-based flow cytometric analysis, cells were fixed and permeabilized with 70% ethanol and then stained with a FITC-conjugated Ki67 antibody (BioLegend, catalog no. 151211). Data were acquired on a BD FACSCelesta cell analyzer, and results were analyzed using FlowJo software (v10). Isotype-matched antibodies served as positive controls for gating.

To evaluate β cell proliferation in pancreatic tissue, the pancreata were fixed and sectioned at 200-μm intervals. Every third section was processed for immunofluorescence staining with antibodies against insulin and Ki67, followed by incubation with respective secondary antibodies. Confocal microscopy was used to capture images. For quantification, approximately 600 insulin-positive cells per animal were counted, with at least five animals in each group. The proportion of proliferation was calculated as the ratio of Ki67^+^ β cells to the total insulin-positive β cells in each section.

For analysis of β cell proliferation in primary mouse islets, a minimum of 20 isolated islets per group were immunostained with primary antibodies targeting insulin and Ki67, followed by incubation with secondary antibodies. The proliferative fraction was calculated as the number of Ki67^+^ β cells relative to the total β cell population within the islets.

### Cell apoptosis detection

Apoptotic β cells in pancreatic tissue sections were identified by dual staining for insulin and TUNEL using a commercial kit (Yeasen, Shanghai, China). The apoptotic index was calculated as the ratio of TUNEL^+^ β cells to the total number of insulin-positive β cells within each section.

To observe β cell apoptosis in primary mouse islets, at least 20 isolated islets per group were costained with insulin and TUNEL. The proportion of apoptotic β cells was quantified as the number of TUNEL^+^ β cells relative to the total β cell population within the islets.

### RNA isolation and qRT-PCR

Total RNA was extracted from cultured cells and sEV preparations using TRIzol reagent (Takara, Shiga, Japan) according to the manufacturer’s protocol. Reverse transcription of miRNAs and mRNAs was performed using commercially available kits (AG, China) following the recommended procedures. Quantitative PCR was conducted in 10 μl of reactions using SYBR Green Master Mix (AG, China) on a Roche LightCycler 480 real-time PCR system (Roche, Basel, Switzerland). The thermal cycling program consisted of an initial denaturation at 95°C for 30 s, followed by 40 cycles of denaturation at 95°C for 5 s and annealing/extension at 60°C for 30 s, with melting curve analysis and cooling. Fluorescence signals were noted at each amplification cycle. Gene expression levels were normalized using *U6* as the internal reference for miRNAs and *ACTB* for mRNAs. For sEV-derived miRNAs, cel-39 was used as an exogenous spike-in control. Absolute miRNA quantification was performed using synthetic miRNA standards of known concentrations, and expression levels were quantified from standard curves.

### sEV miRNA sequencing

Murine pancreatic islets were isolated as previously described (*47*) and cultured in serum-free medium for 48 hours. Conditioned media were collected for sEV isolation. Total RNA from sEVs was extracted using the miRNeasy Mini Kit (QIAGEN, USA), and 5 ng of RNA was used for miRNA library construction with the NEBNext Ultra Small RNA Library Prep Kit (New England Biolabs, USA). High-throughput sequencing was performed on an Illumina NovaSeq 6000 platform. Sequencing and downstream bioinformatic analyses were carried out by Hangzhou KAITAI Biotechnology Co. Ltd. (China). The raw sequencing data have been deposited in the Gene Expression Omnibus (GEO) database under accession number GSE307474 (www.ncbi.nlm.nih.gov/geo/query/acc.cgi?acc=GSE307474).

### Plasmid construction, transfection, and viral infection

The coding sequences and 3′UTRs of *Klf9* (NM_010638), *E2f6* (NM_033270.2), and *Mnt3* (NM_010813) were amplified from full-length MIN6 cDNA by PCR and cloned into the PLVX-IRES-ZsGreen vector. Primer sequences are provided in table S5. Single-guide RNAs (sgRNAs) targeting *Dicer*, *F11r*, *Stxbp*, and *Vamp2* were inserted into the linearized lentiCRISPRv2-puro vector following established protocols (*48*). Vector was designed using Bsm BI, and *sgRNA* sequences are listed in table S5. Transient transfections were conducted using Lipofectamine 2000 (Invitrogen) according to the manufacturer’s instructions. To generate stable KD or overexpression cell lines, lentiCRISPRv2 or PLVX-IRES-ZsGreen constructs were cotransfected with packaging and envelope plasmids into human embryonic kidney (HEK) 293T cells to produce lentiviral particles. Target cells were infected and selected using puromycin (MedChemExpress, USA) or fluorescence-activated cell sorting.

### Western blot analysis

MIN6 cells or MSCs were lysed in radioimmunoprecipitation assay buffer (Beyotime, Shanghai, China) supplemented with 1% phenylmethylsulfonyl fluoride on ice for 30 min. Protein concentrations were determined using a BCA assay, followed by heat denaturation. Equal amounts of protein were separated by SDS–polyacrylamide gel electrophoresis and transferred onto polyvinylidene difluoride membranes (0.22 μm). Membranes were blocked for 1 hour and incubated with primary antibodies at 4°C overnight. Protein bands were visualized using a Tanon 3500 imaging system, and densitometric analysis was performed with Fiji software.

### Islet mass analysis

For quantification of islet mass, pancreata were fixed in formalin, embedded in paraffin, and sectioned at 200-μm intervals (six sections per mouse). Tissue sections were stained with hematoxylin and eosin and digitally scanned using the NDP View system (Hamamatsu, Japan). At least five nonadjacent sections per pancreas were analyzed. Islet mass was calculated as the ratio of total islet area to total pancreatic area.

### Immunofluorescence analysis

Paraffin-embedded pancreatic sections or fixed cultured cells were incubated with primary antibodies, followed by incubation with Alexa Fluor–conjugated secondary antibodies, including goat anti-rabbit immunoglobulin G (IgG) (Alexa Fluor 488, Abcam, ab150077), goat anti-mouse IgG (Alexa Fluor 594, Abcam, ab150116), or goat anti-mouse IgG (Alexa Fluor 647, Abcam, ab150115). Fluorescent images were captured using a Zeiss LSM800 confocal laser scanning microscope (Zeiss, Oberkochen, Germany) and analyzed using Fiji (*49*).

### Luciferase reporter assay

To validate miRNA-target interactions, the 3′UTR containing either *Klf9*-WT or *Klf9*-MUT was cloned downstream of the firefly luciferase coding region. *Klf9*-WT or *Klf9*-MUT constructs were cotransfected with *miR-151* mimics and a *Renilla* luciferase control plasmid.

For promoter activity analysis, the promoter regions of *Wnt3a* and *Ccnd1* were inserted into luciferase reporter vectors and cotransfected with a *Klf9* overexpression plasmid. Cells were harvested 24 hours posttransfection, and luciferase activity was measured using a dual-luciferase reporter assay system (Promega). Firefly luciferase signals were normalized to *Renilla* luciferase activity.

### RNA binding protein immunoprecipitation assay

RNA immunoprecipitation assays were performed as previously described (*50*). Approximately 1 × 10^8^ cells were collected and lysed in 100 μl of NP-40 buffer supplemented with protease and ribonuclease inhibitors. Cell lysates were incubated with IgG- or Ago2-conjugated beads at 4°C under continuous rotation. After centrifugation to remove unbound material, bead-associated complexes were treated with proteinase K. Immunoprecipitated RNAs were extracted with phenol-chloroform and analyzed by qRT-PCR.

### Statistical analysis

All experiments were performed with at least three biological replicates. Experimental groups were compared using Student’s *t* test. Multiple groups were compared using Dunn’s post hoc test after one-way analysis of variance (ANOVA), while Fisher’s least significant difference was used following two-way ANOVA. Significance levels were set at *P* < 0.05, *P* < 0.01, and *P* < 0.001. All statistical analyses were performed using GraphPad Prism 9, and data are shown as means ± SD.
