# Supplementary material for: Mesenchymal stem cells receive adaptive islet–derived miR-151–containing sEVs to promote β cell compensation in obesity
Source: Sci Adv. 2026 Jul 17;12(29):eadu4196. doi: 10.1126/sciadv.adu4196 (PMC13378542; doi:10.1126/sciadv.adu4196)
Supplement: Supplementary file 1 — Figs. S1 to S7 Tables S1 to S6 Legend for data S1 [file sciadv.adu4196_sm.pdf]

Science Advances
AAAS

Supplementary Materials for

Mesenchymal stem cells receive adaptive islet–derived *miR-151*–containing sEVs to promote β cell compensation in obesity

Xinwei Guo *et al.*

Corresponding author: Wei Tang, drtangwei@njmu.edu.cn; Yue Liu, 20220105@nxmu.edu.cn; Liang Jin, ljstemcell@cpu.edu.cn

Sci. Adv. **12**, eadu4196 (2026)
DOI: 10.1126/sciadv.adu4196

The PDF file includes:

Figs. S1 to S7
Tables S1 to S6
Legend for data S1

Other Supplementary Material for this manuscript includes the following:

Data S1

Supplementary Text

Fig. S1.

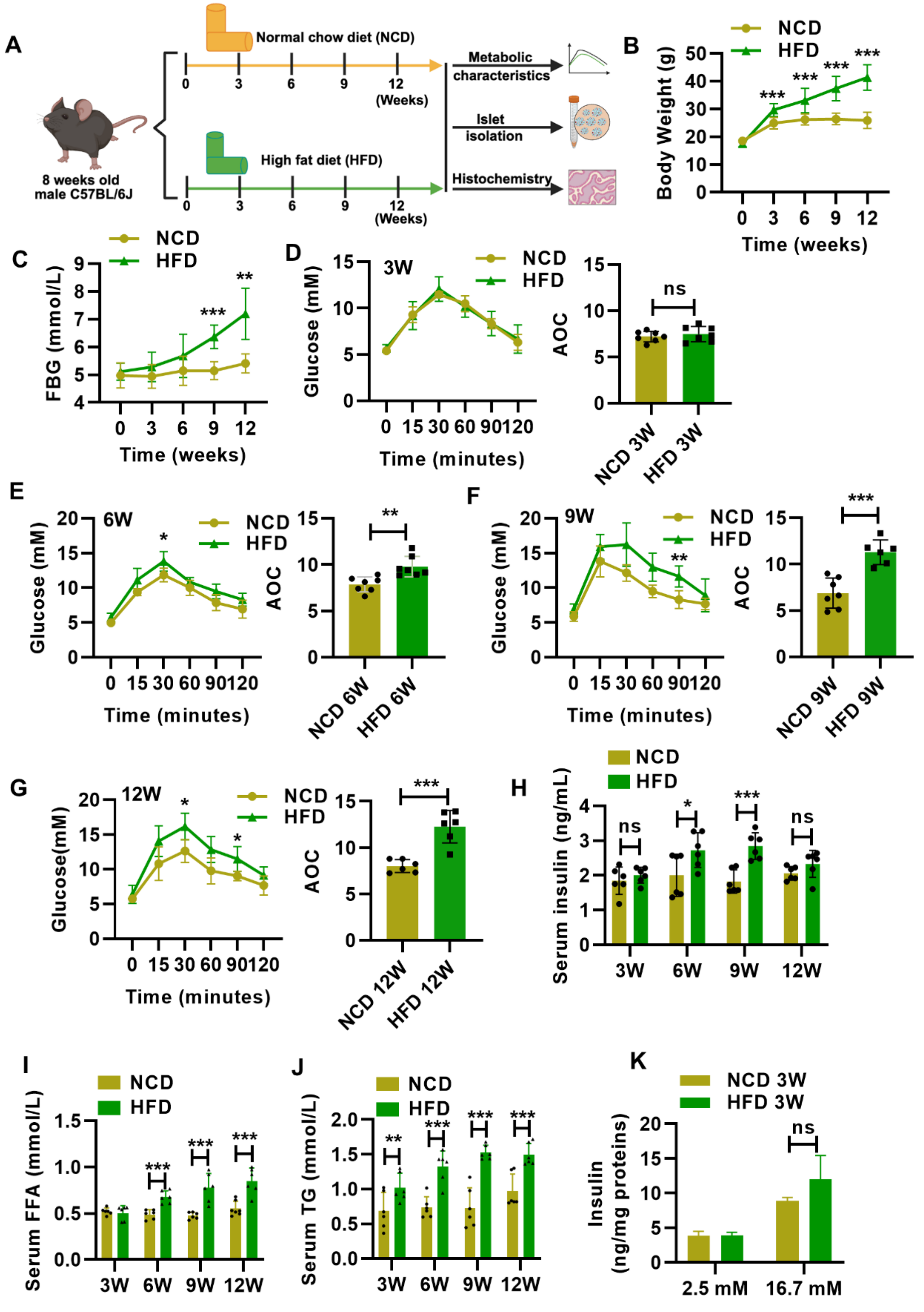

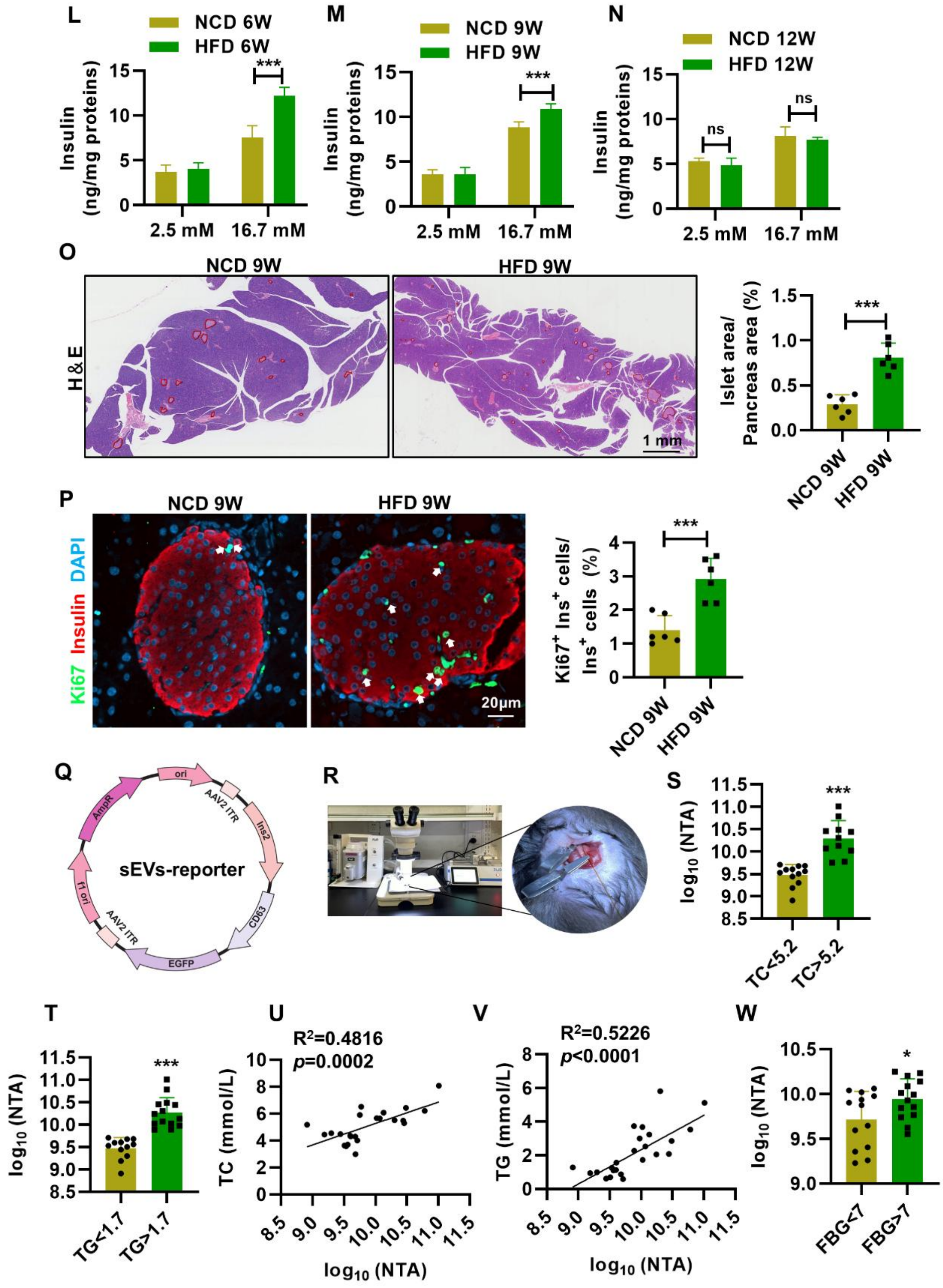

Supplementary Figure 1. The sEVs secretion is elevated in adaptive islet β-cell. *A*: 8-week-old C57BL/6J mice were fed with normal chow diet (NCD) or high fat diet (HFD) for 12 weeks. Metabolic characters were measured at the indicated time. The icon or graphical images were

created in BioRender. Guo, X. (2026) https://BioRender.com/m618tu3. ***B-C***: Body weight *(B)* and fasting blood glucose (FBG) *(C)* were recorded every 3 weeks (n=6 mice/group). ***D-G***: IPGTT was performed on mice exposed to an HFD diet for 3 weeks *(D)*, 6 weeks *(E)*, 9 weeks *(F),* and 12 weeks *(G)*. The area of curve (AOC) of blood glucose level was calculated (n=6 mice/group). ***H-J***: Serum samples were collected from mice exposed to an HFD diet for 3 weeks, 6 weeks, 9 weeks, and 12 weeks. The levels of serum insulin *(H)*, FFA *(I)*, and TG *(J)* were measured (n=6 mice/group). ***K-N***: GSIS assay was performed on islets isolated from mice exposed to an HFD diet for 3 weeks *(K)*, 6 weeks *(L)*, 9 weeks *(M)*, and 12 weeks *(N)* (n=3 mice/group). ***O***: Representative images and statistical analysis of pancreatic section in mice exposed to an HFD diet for 9 weeks (n=6 mice/group). Magnification, ×2; scale bar, 1 mm. ***P***: Representative images and statistical analysis of $Ki67^+$ β-cells in mice exposed to an HFD diet for 9 weeks (n=6 mice/group). Magnification, ×20; scale bar, 20 μm. ***Q***: The diagram of Ins2-CD63-EGFP AAV8 plasmid. CD63-EGFP fusion protein is expressed under the control of ins2 promoter. The plasmid was then subjected to a packaging procedure to produce Ins2-CD63-EGFP AAV8 viruses. ***R***: The Ins2-CD63-EGFP AAV8 viruses were injected into C57BL/6J mice aged 8 weeks via pancreatic ductal infusion. ***S***: NTA analysis of serum sEVs derived from normal volunteers and hypercholesterolemia patients (n = 23). ***T***: NTA analysis of serum sEVs derived from normal volunteers and hypertriglyceride patients (n = 26). ***U***: Correlation between particles number of serum sEVs and serum total cholesterol (TC) level (n = 23). ***V***: Correlation between particles number of serum sEVs and serum triglyceride (TG) level (n = 26). ***W***: NTA analysis of serum sEVs derived from normal volunteers and hyperglycemia patients (n = 27). Data are presented as mean ± SD. **p*<0.05, ***p*<0.01, ****p*<0.001 by Student's t test *(K-P, S-T, W)* or two-way ANOVA*(B-J)*.

Fig. S2.

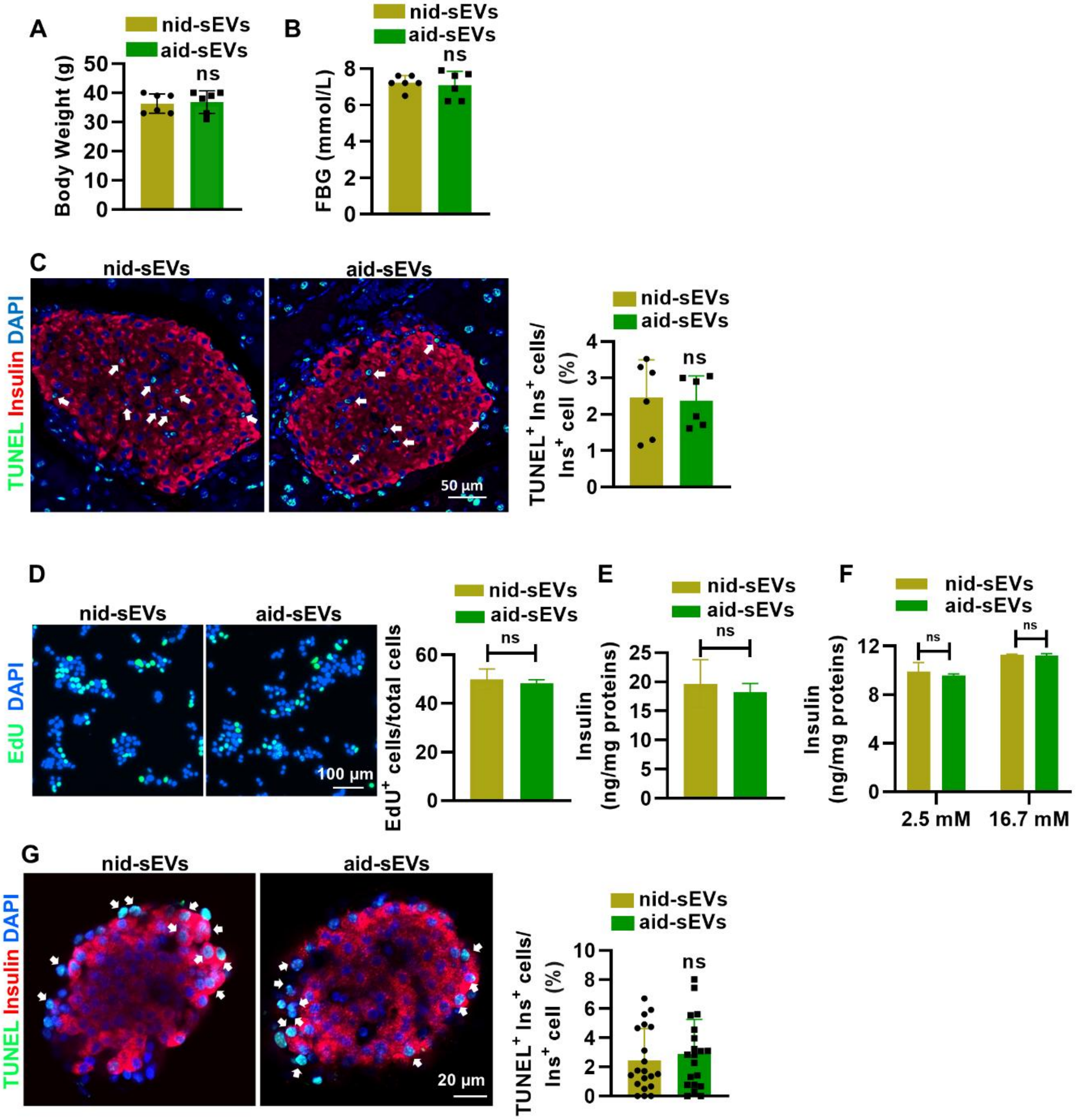

Supplementary Figure 2. Aid-sEVs promote the development of islet β-cell compensation. 8 weeks old male C57BL/6J mice were fed with high fat diet (HFD) for 6 weeks. Subsequently, mice were injected with nid-sEVs and aid-sEVs intravenously (twice a week, 4 weeks in total). All analyses were performed 2 weeks post-treatment termination. ***A-B***: Body weight *(A)* and fasting blood glucose *(B)* of mice were measured before sacrifices (n=6 mice/group). ***C***: Representative images and statistical analysis of TUNEL$^+$ β-cells in mice treated with nid-sEVs and aid-sEVs (n=6 mice/group). Magnification, ×20; scale bar, 20 μm. ***D-F***: MIN6 cells were

treated with nid-sEVs and aid-sEVs for 24 h. EdU (green) immunofluorescence *(D)*, Magnification, ×10; scale bar, 100 μm, insulin level in medium *(E)*, and GSIS assay *(F)* were performed (n=3/group). ***G***: Primary islets were isolated and treated with nid-sEVs or aid-sEVs for 24 h, then the islets were stained with TUNEL (green) and insulin (red). At least 20 islets were analyzed in each group. Magnification, ×20; scale bar, 20 μm. Data are presented as mean ± SD. **p*<0.05, ***p*<0.01, ****p*<0.001 by Student's t test.

Fig. S3.

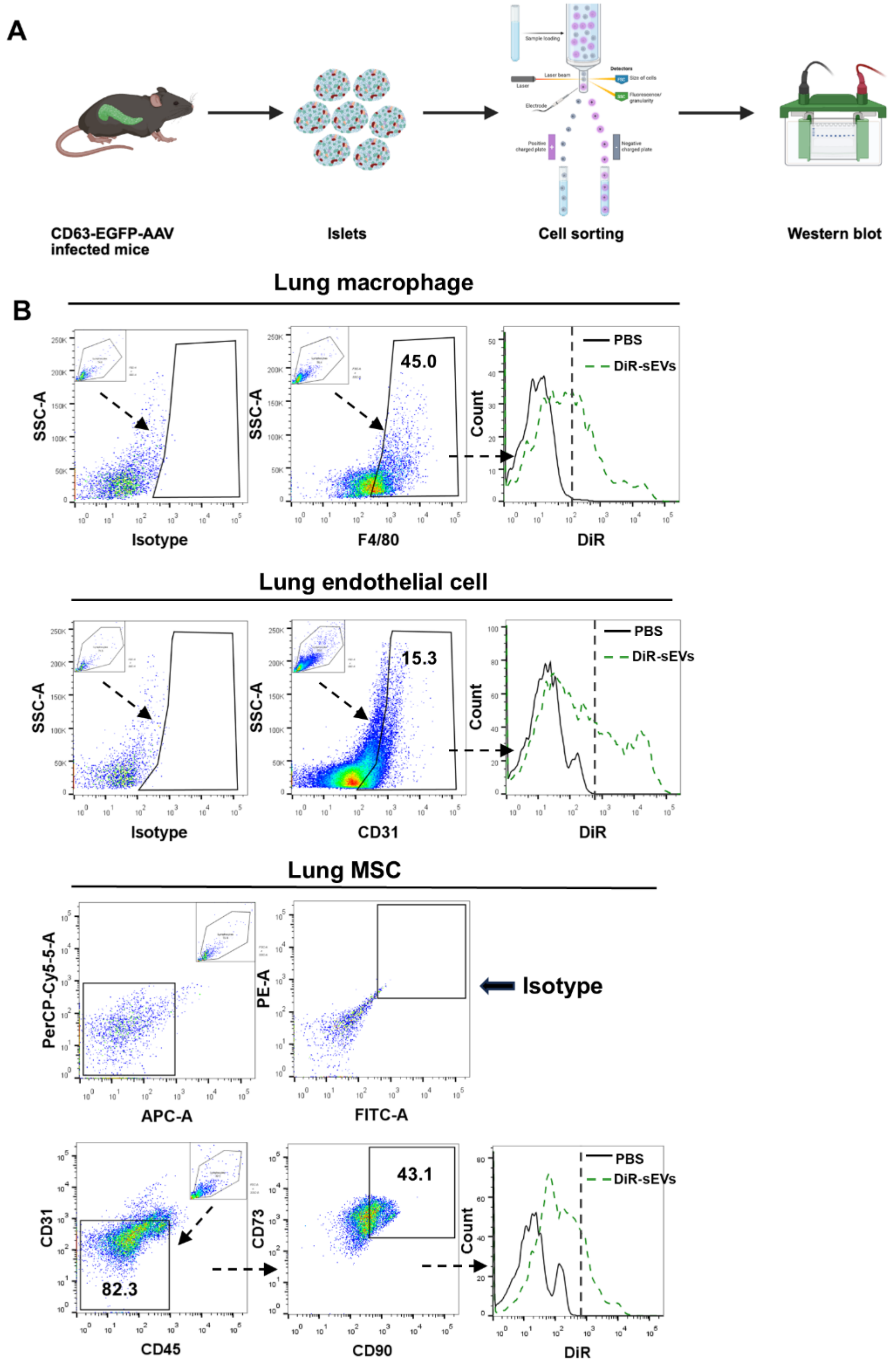
A
CD63-EGFP-AAV infected mice
Islets
Cell sorting
Western blot
B
Lung macrophage
SSC-A
Isotype
F4/80
45.0
Count
DiR
PBS
DiR-sEVs
Lung endothelial cell
CD31
15.3
Lung MSC
PerCP-Cy5-5-A
APC-A
PE-A
FITC-A
Isotype
CD31
CD45
82.3
CD73
CD90
43.1

C

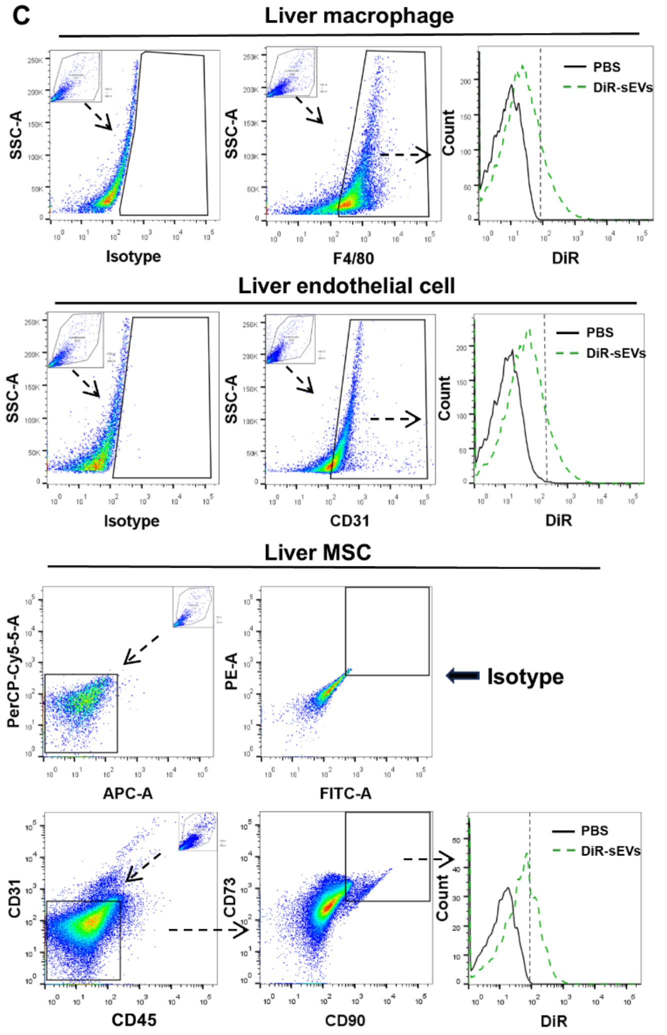

Liver macrophage
SSC-A
Isotype
F4/80
Count
DiR
PBS
DiR-sEVs
Liver endothelial cell
CD31
Liver MSC
PerCP-Cy5-5-A
APC-A
PE-A
FITC-A
Isotype
CD45
CD73
CD90

D

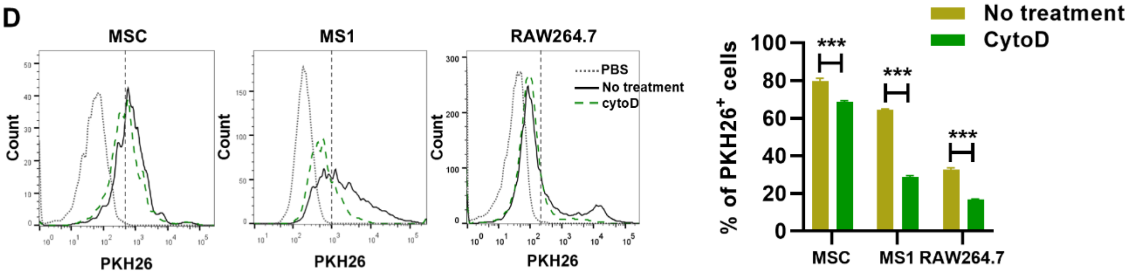

MSC
MS1
RAW264.7
Count
PKH26
PBS
No treatment
cytoD
CytoD
% of PKH26+ cells
100
80
60
40
20
0
***

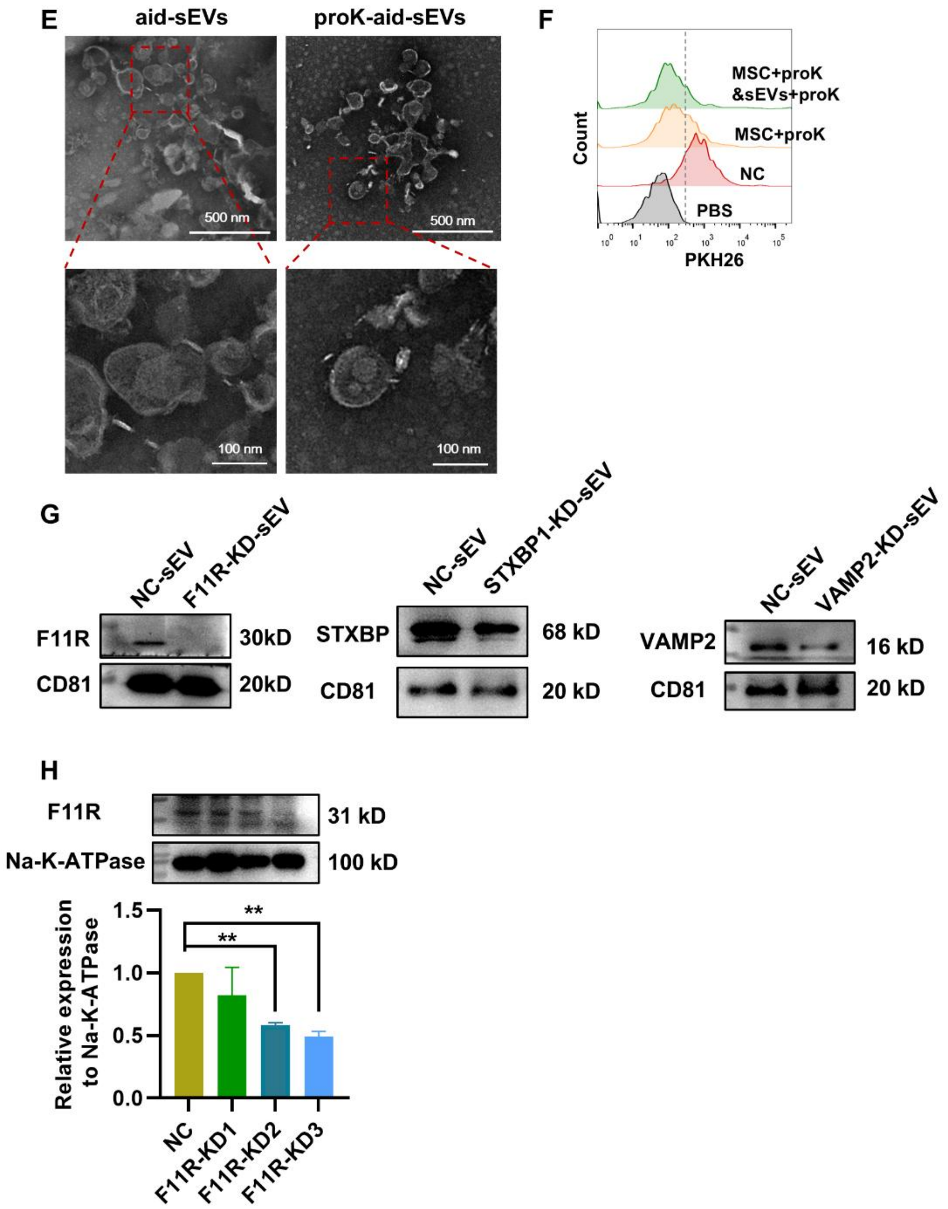

Supplementary Figure 3. Aid-sEVs uptake in recipient cells is partially dependent on F11R. ***A***: Schematic diagram showing detection of EGFP in MSCs, macrophages and endothelial cells in mice injected with Ins2-CD63-EGFP AAV viruses. The icon or graphical images were created in BioRender. Guo, X. (2026) https://BioRender.com/m618tu3. ***B***: The internalization of DiR-labelled sEVs by MSCs, macrophages and endothelial cells in the lungs of C57BL/6J mice administered with DiR-labelled sEVs intravenously. Isotype and PBS were used as control

(n=3/group). ***C***: The internalization of DiR-labelled sEVs by MSCs, macrophages and endothelial cells in the livers of C57BL/6J mice administered with DiR-labelled sEVs intravenously. Isotype and PBS were used as control (n=3/group). ***D:*** MSC, MS1 and RAW264.7 cells were preincubated with 5 μM cytochalasin D and then treated with PKH26-labelled sEVs. Flow cytometry was used to detect the internalization of PKH26-labelled sEVs by these cells. Cells incubated with PBS alone were used as control (n=3/group). ***E***: TEM images of proteinase K treated aid-sEVs. Scale bar, 500 nm, 100 nm. ***F***: MSC and aid-sEVs were treated with proteinase K. Flow cytometry was used to detect the internalization of PKH26-labelled sEVs by these cells. Cells incubated with PBS alone were used as control (n=3/group). ***G:*** The knockdown efficiency of F11R, STXBP and VAMP2 in respective sEVs. CD81 was used as control. ***H***: The knockdown efficiency of F11R in MSCs. Na-K-ATPase was used as control (n=3/group). Data are presented as mean $\pm$ SD. *$p<0.05$, **$p<0.01$, ***$p<0.001$ by Student's t test (*D*), and one-way ANOVA (*H*).

Fig. S4

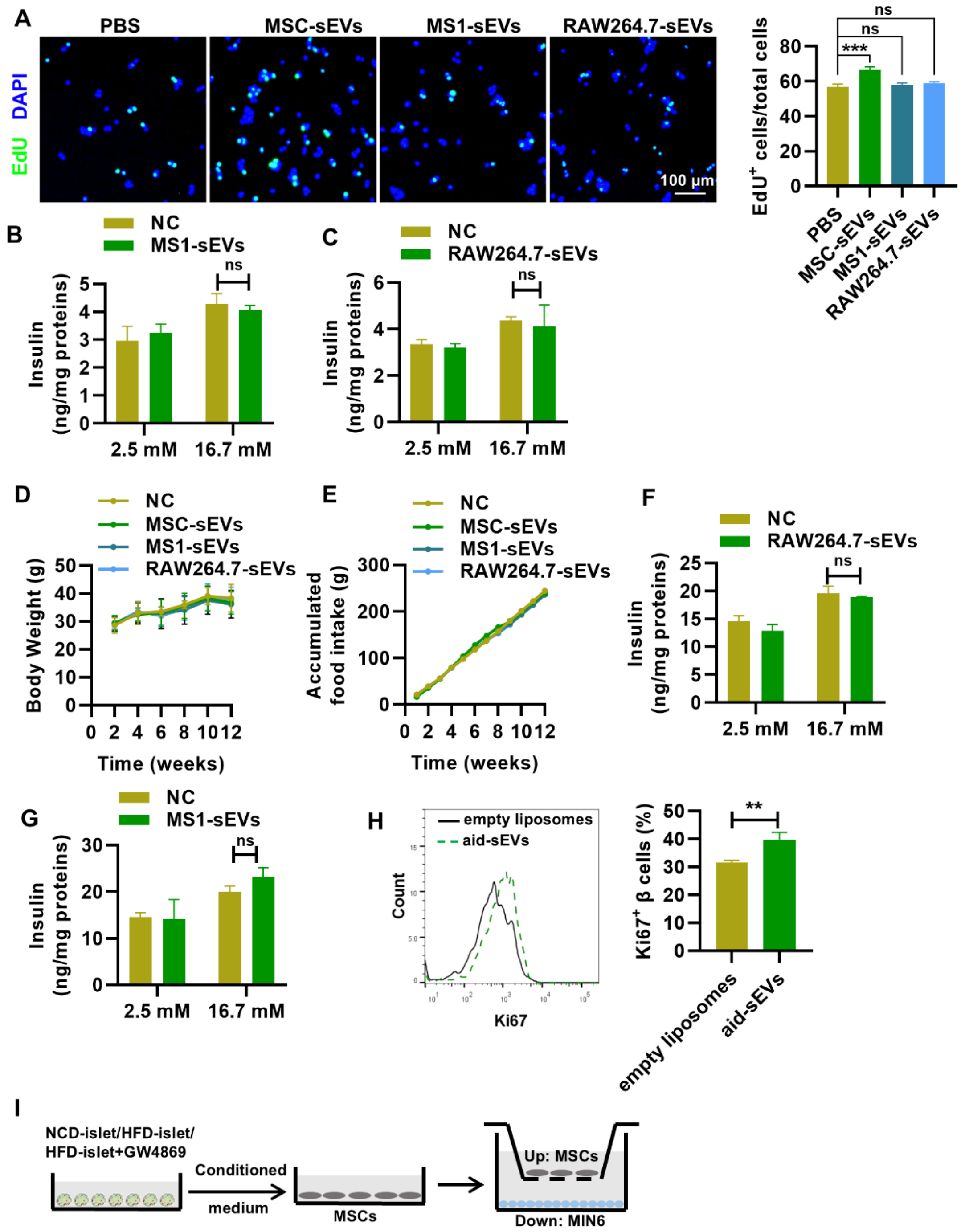

A
PBS
MSC-sEVs
MS1-sEVs
RAW264.7-sEVs
EdU DAPI
100 μm
EdU+ cells/total cells
ns
ns
***
PBS
MSC-sEVs
MS1-sEVs
RAW264.7-sEVs
B
NC
MS1-sEVs
ns
Insulin (ng/mg proteins)
2.5 mM
16.7 mM
C
NC
RAW264.7-sEVs
ns
Insulin (ng/mg proteins)
2.5 mM
16.7 mM
D
NC
MSC-sEVs
MS1-sEVs
RAW264.7-sEVs
Body Weight (g)
Time (weeks)
E
NC
MSC-sEVs
MS1-sEVs
RAW264.7-sEVs
Accumulated food intake (g)
Time (weeks)
F
NC
RAW264.7-sEVs
ns
Insulin (ng/mg proteins)
2.5 mM
16.7 mM
G
NC
MS1-sEVs
ns
Insulin (ng/mg proteins)
2.5 mM
16.7 mM
H
empty liposomes
aid-sEVs
Count
Ki67
Ki67+ β cells (%)
**
empty liposomes
aid-sEVs
I
NCD-islet/HFD-islet/
HFD-islet+GW4869
Conditioned
medium
MSCs
Up: MSCs
Down: MIN6

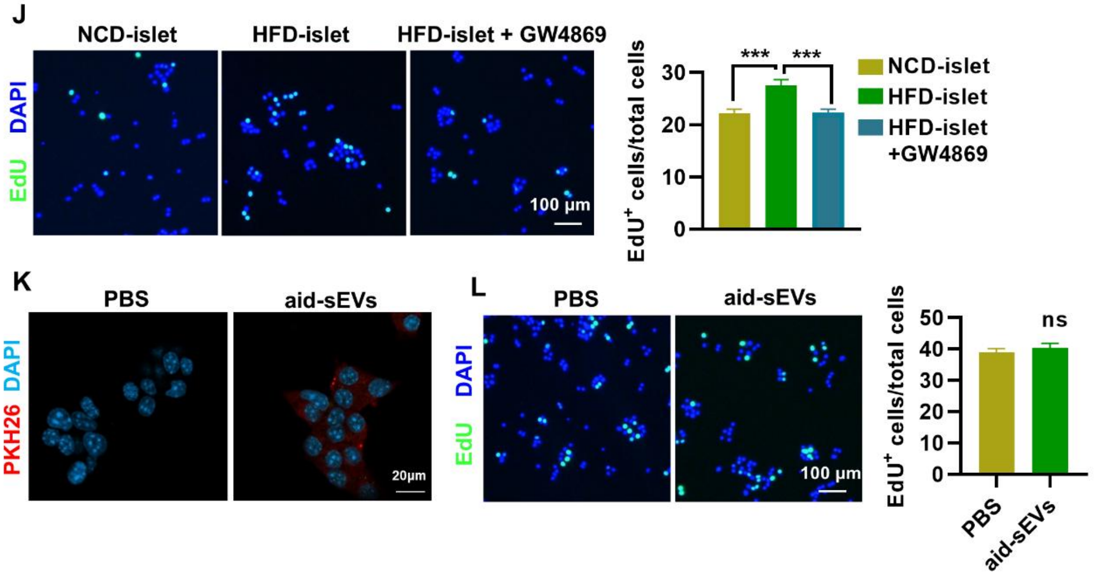

Supplementary Figure 4. MSCs received the aid-sEVs are effector cells to promote β-cell compensation. ***A***: EdU assay was performed to detect the proliferation of MIN6 cells co-cultured with PBS, MSC-sEVs, MS1-sEVs and RAW264.7-sEVs. Magnification, ×10; scale bar, 100 µm. (n=3/group). ***B-C***: GSIS of islets treated with MS1-sEVs *(B)* and RAW264.7-sEVs *(C)* (n=3/group). ***D-E***: Body weight *(D)* and food intake *(E)* were measured in PBS, MSC-sEVs, MS1-sEVs and RAW264.7-sEVs injected mice (n=6 mice/group). ***F-G***: GSIS of islets isolated from RAW264.7-sEVs *(F)* and MS1-sEVs *(G)* injected mice (n=3/group). ***H***: Ki67 staining was performed to detect the proliferation of MSCs treated with aid-sEVs (n=3/group). ***I-J***: The conditioned medium from NCD-islet, HFD-islet, or HFD-islet treated with GW4869 (10 µg/mL) were collected and incubated with MSC for 24 h. MIN6 cells were co-cultured with relative MSCs and subjected to EdU assay. Magnification, ×10; scale bar, 100 µm. (n=3/group). The icon or graphical images were created in BioRender. Guo, X. (2026) https://BioRender.com/bw6f83d ***K***: The internalization of PKH26-labelled sEVs by MIN6 cells. After adding PKH26-labelled aid-sEVs to the medium of MIN6 cells for 24 h, images were captured by confocal microscope. PBS was used as control. Magnification, ×20; scale bar, 20 µm. ***L***: EdU assay was performed to detect the proliferation of MIN6 cells treated with aid-sEVs. Magnification, ×10; scale bar, 100 µm. (n=3/group). Data are presented as mean ± SD. *p<0.05, **p<0.01, ***p<0.001 by Student's t test *(B, C, F-H, L)* or one-way ANOVA *(A, J)*.

Fig. S5

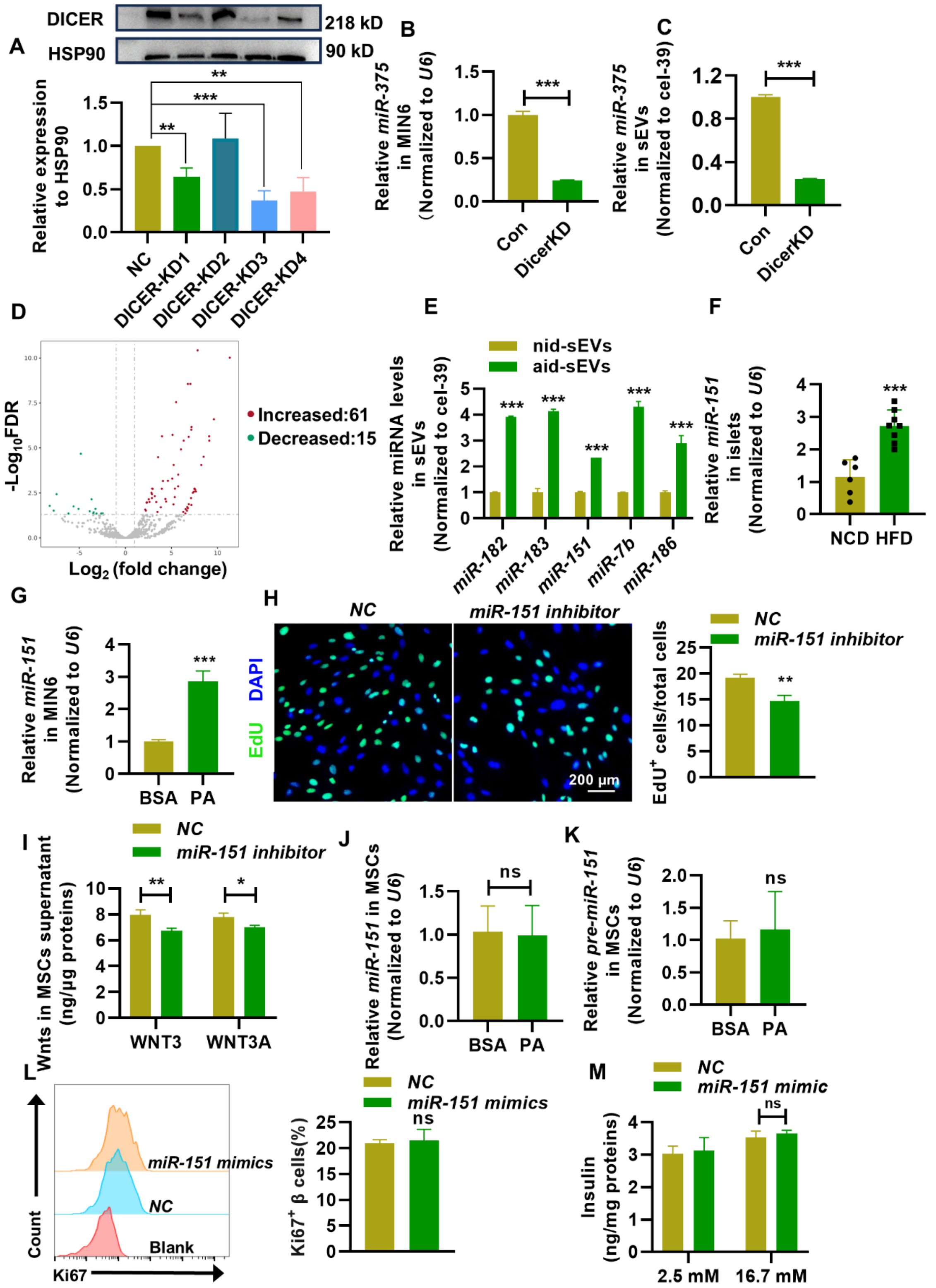

A
DICER
HSP90
218 kD
90 kD
Relative expression to HSP90
NC
DICER-KD1
DICER-KD2
DICER-KD3
DICER-KD4
B
Relative miR-375 in MIN6 (Normalized to U6)
Con
DicerKD
C
Relative miR-375 in sEVs (Normalized to cel-39)
D
-Log10FDR
Log2 (fold change)
Increased:61
Decreased:15
E
nid-sEVs
aid-sEVs
Relative miRNA levels in sEVs (Normalized to cel-39)
miR-182
miR-183
miR-151
miR-7b
miR-186
F
Relative miR-151 in islets (Normalized to U6)
NCD
HFD
G
Relative miR-151 in MIN6 (Normalized to U6)
BSA
PA
H
NC
miR-151 inhibitor
DAPI
EdU
200 μm
EdU+ cells/total cells
I
Wnts in MSCs supernatant (ng/μg proteins)
WNT3
WNT3A
J
Relative miR-151 in MSCs (Normalized to U6)
K
Relative pre-miR-151 in MSCs (Normalized to U6)
L
miR-151 mimics
Blank
Count
Ki67
Ki67+ β cells(%)
M
miR-151 mimic
Insulin (ng/mg proteins)
2.5 mM
16.7 mM

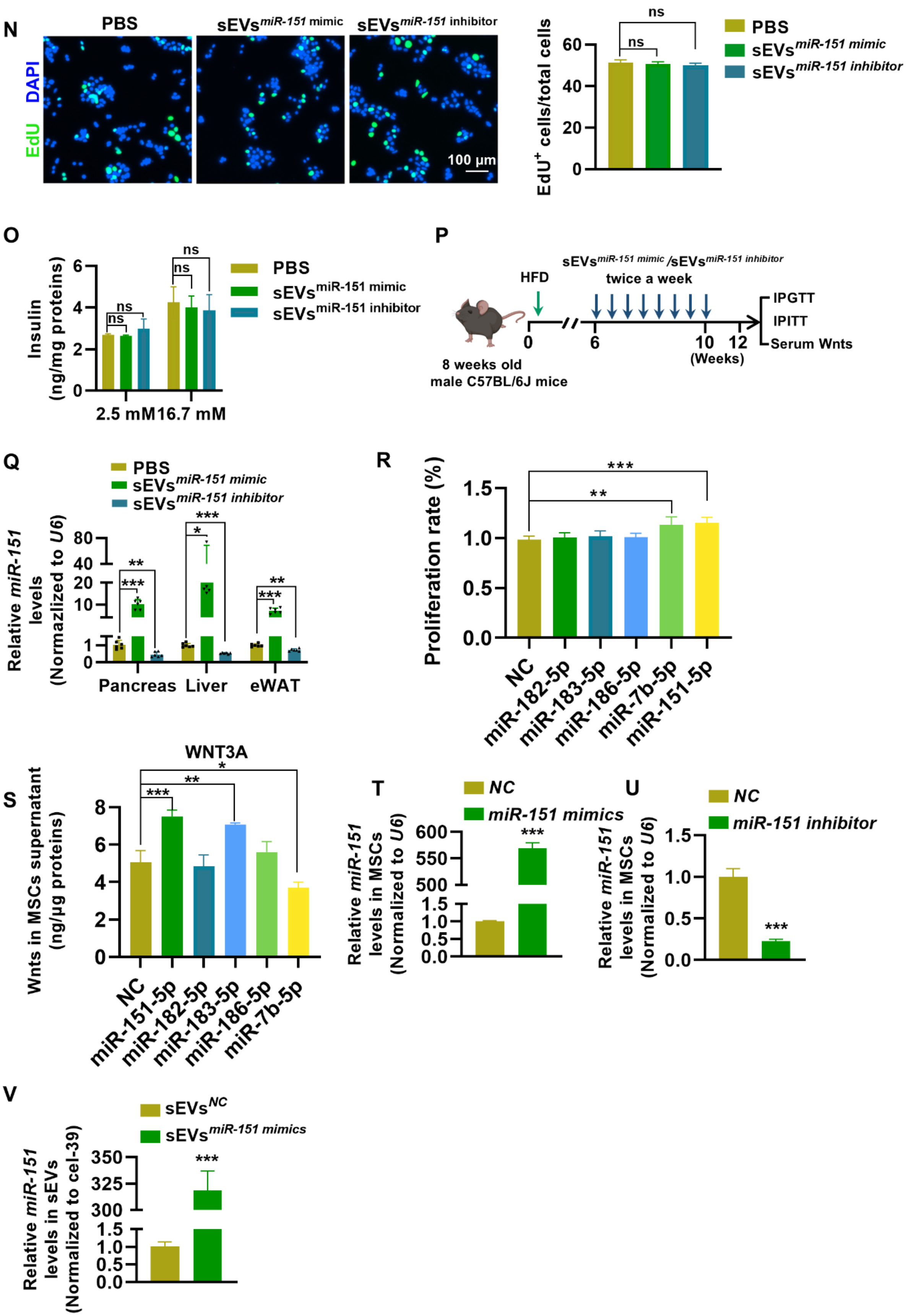
N
PBS
sEVs miR-151 mimic
sEVs miR-151 inhibitor
EdU DAPI
100 μm
EdU+ cells/total cells
ns
ns
O
Insulin (ng/mg proteins)
2.5 mM
16.7 mM
PBS
sEVs miR-151 mimic
sEVs miR-151 inhibitor
P
HFD
sEVs miR-151 mimic /sEVs miR-151 inhibitor
twice a week
0
6
10
12
(Weeks)
8 weeks old
male C57BL/6J mice
IPGTT
IPITT
Serum Wnts
Q
PBS
sEVs miR-151 mimic
sEVs miR-151 inhibitor
Relative miR-151 levels (Normazlized to U6)
Pancreas
Liver
eWAT
R
Proliferation rate (%)
NC
miR-182-5p
miR-183-5p
miR-186-5p
miR-7b-5p
miR-151-5p
S
WNT3A
Wnts in MSCs supernatant (ng/μg proteins)
NC
miR-151-5p
miR-182-5p
miR-183-5p
miR-186-5p
miR-7b-5p
T
NC
miR-151 mimics
Relative miR-151 levels in MSCs (Normalized to U6)
U
NC
miR-151 inhibitor
Relative miR-151 levels in MSCs (Normalized to U6)
V
sEVs NC
sEVs miR-151 mimics
Relative miR-151 levels in sEVs (Normalized to cel-39)

Supplementary Figure 5. *miR-151* is responsible for the aid-sEVs effects on β-cell adaption. ***A***: The knockdown efficiency of DICER in MIN6 cells. HSP90 was used as control (n=3). ***B-C***: qRT-PCR was performed to detect the expression level of *miR-375* in DicerKD and control MIN6 cells *(B)* and respective sEVs *(C)* (n=3/group). ***D***: Volcano plot of differentially expressed miRNAs in nid-sEVs and aid-sEVs. ***E***: qRT-PCR was performed to detect the relative expression levels of top 5 most abundant differentially expressed miRNAs in nid-sEVs and aid-sEVs (n=3/group). ***F-G***: qRT-PCR was performed to detect the expression level of *miR-151* in islets *(F)* (n⩾6 mice/group) and MIN6 cells *(G)* (n=3/group). ***H***: EdU assay was performed to detect the proliferation of MSCs transfected with *NC* and *miR-151 inhibitor* (n=3/group). Magnification, ×10; scale bar, 200 μm. ***I***: The levels of WNT3 and WNT3A in supernatant of MSCs transfected with *NC* and *miR-151 inhibitor* (n=3/group). ***J-K***: qRT-PCR was performed to detect the expression levels of *miR-151 (J)* and *pre-miR-151 (K)* in MSCs treated with PA (n=3/group). ***L***: Ki67 staining was performed to detect the proliferation of MSCs transfected with *miR-151 mimic* (n=3/group). ***M***: GSIS of MIN6 cells transfected with *miR-151 mimic* (n=3/group). ***N***: EdU assay was performed to detect the proliferation of MIN6 cells treated with PBS, $sEVs^{\textit{miR-151}\text{ mimic}}$ and $sEVs^{\textit{miR-151}\text{ inhibitor}}$ (n=3/group). Magnification, ×10; scale bar, 100 μm. ***O***: GSIS of MIN6 cells treated with PBS, $sEVs^{\textit{miR-151}\text{ mimic}}$ and $sEVs^{\textit{miR-151}\text{ inhibitor}}$ (n=3/group). ***P***: Flowchart of the *in vivo* experiment designed for detecting the function of $sEVs^{\textit{miR-151}\text{ mimic}}$ on β-cell. The icon or graphical images were created in BioRender. Guo, X. (2026) https://BioRender.com/bw6f83d**.** ***Q***: qRT-PCR was performed to detect the delivery efficiency of sEVs (n=6 mice/group). ***R***: The proliferation rate of MSCs transfected with different miRNAs (n=3/group). ***S***: The levels of WNT3A in supernatant of MSCs transfected with different miRNAs (n=3/group). ***T-U***: qRT-PCR was performed to detect the transfection efficiency of *miR-151* mimics *(T)* and inhibitor *(U)* in MSCs (n=3/group). ***V***: qRT-PCR was performed to detect the overexpression of *miR-151* in sEVs (n=3/group). Data are presented as mean ± SD. *p<0.05, **p<0.01, ***p<0.001 by Student's t test *(B, C, E-M, T-V)* or one-way ANOVA *(A, N, O, Q, R-S)*.

Fig.S6

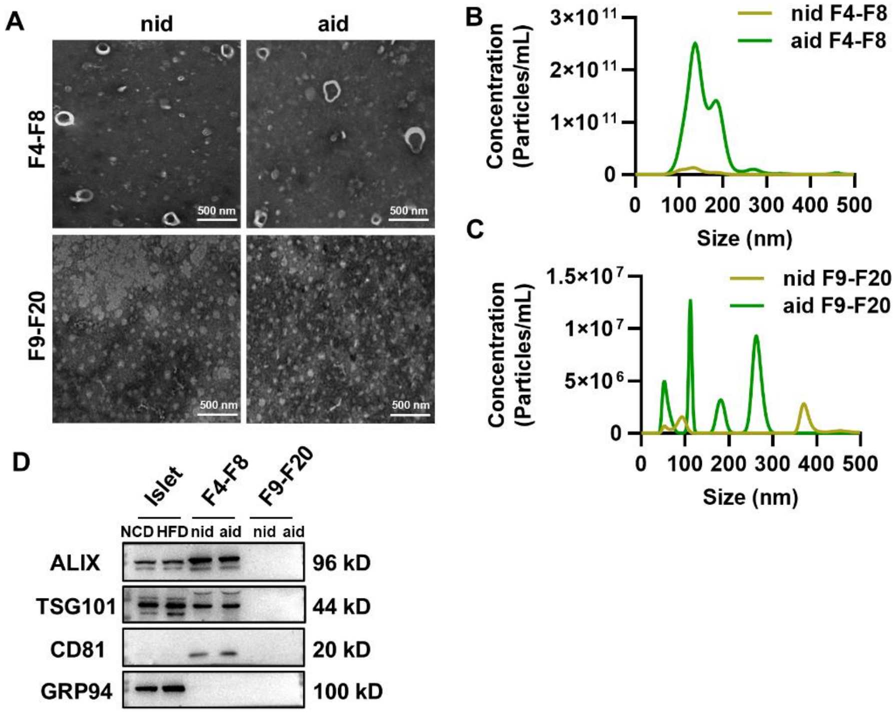

Supplementary Figure 6. Purified aid-sEVs promote islet compensation. ***A:*** TEM imaging of F4-8 and F9-20 in different groups (Scale bar, 500 nm). ***B-C:*** NTA analysis of F4-8 and F9-20 in different groups (n=3/group). ***D:*** sEVs markers were detected in F4-8 and F9-20 of different groups.

.

Fig.S7

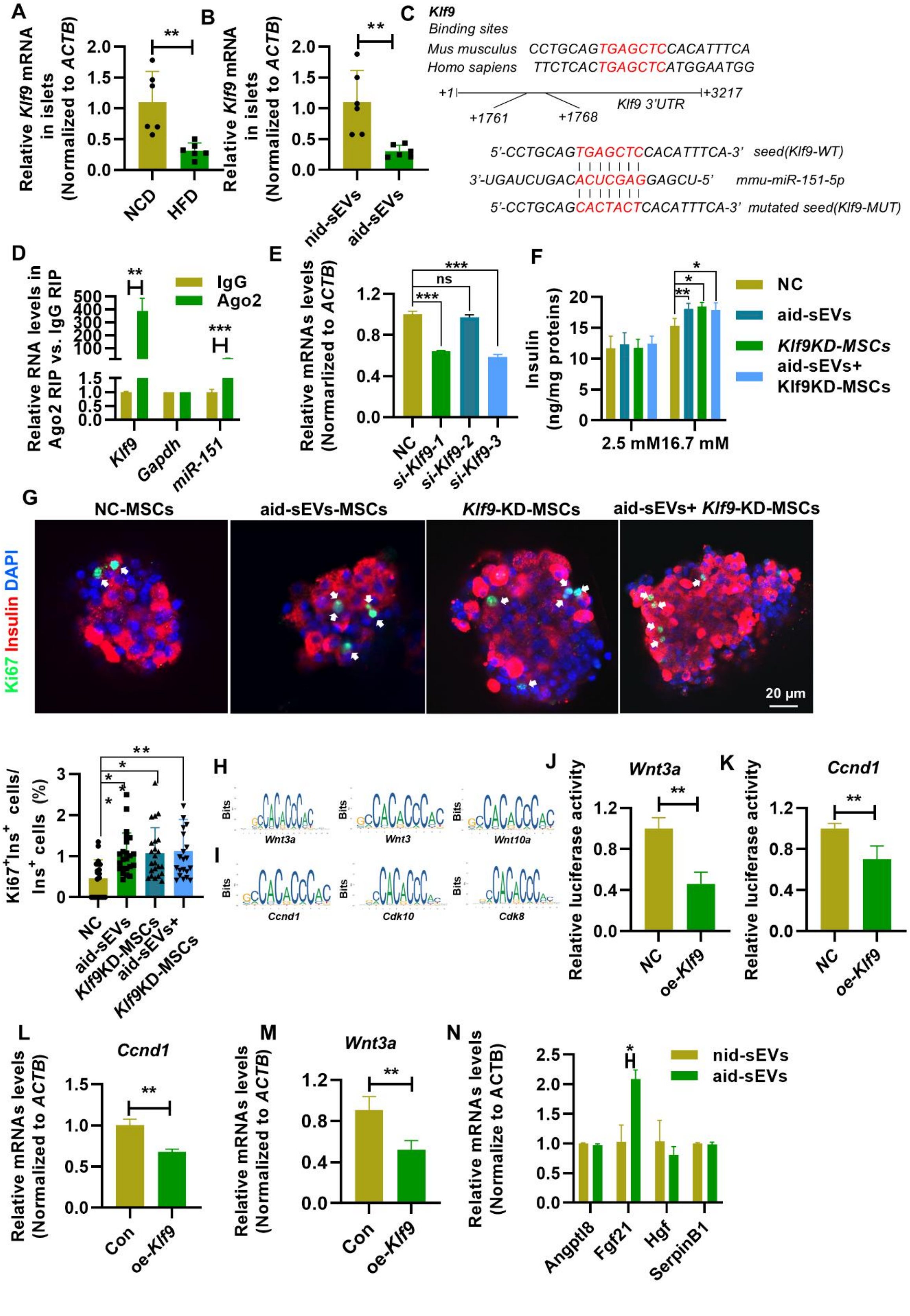
A
Relative Klf9 mRNA in islets (Normalized to ACTB)
NCD
HFD
B
nid-sEVs
aid-sEVs
C
Klf9
Binding sites
Mus musculus CCTGCAGTGAGCTCCACATTTCA
Homo sapiens TTCTCACTGAGCTCATGGAATGG
+1
+3217
+1761
+1768
Klf9 3'UTR
5'-CCTGCAGTGAGCTCCACATTTCA-3' seed(Klf9-WT)
3'-UGAUCUGACACUCGAGGAGCU-5' mmu-miR-151-5p
5'-CCTGCAGCACTACTCACATTTCA-3' mutated seed(Klf9-MUT)
D
Relative RNA levels in Ago2 RIP vs. IgG RIP
IgG
Ago2
Klf9
Gapdh
miR-151
E
Relative mRNAs levels (Normarlized to ACTB)
NC
si-Klf9-1
si-Klf9-2
si-Klf9-3
ns
F
Insulin (ng/mg proteins)
2.5 mM
16.7 mM
NC
aid-sEVs
Klf9KD-MSCs
aid-sEVs+ Klf9KD-MSCs
G
NC-MSCs
aid-sEVs-MSCs
Klf9-KD-MSCs
aid-sEVs+ Klf9-KD-MSCs
Ki67 Insulin DAPI
20 μm
Ki67+Ins+ cells/ Ins+ cells (%)
NC
aid-sEVs
Klf9KD-MSCs
aid-sEVs+ Klf9KD-MSCs
H
Wnt3a
Wnt3
Wnt10a
I
Ccnd1
Cdk10
Cdk8
J
Wnt3a
Relative luciferase activity
NC
oe-Klf9
K
Ccnd1
Relative luciferase activity
NC
oe-Klf9
L
Ccnd1
Relative mRNAs levels (Normalized to ACTB)
Con
oe-Klf9
M
Wnt3a
Relative mRNAs levels (Normalized to ACTB)
Con
oe-Klf9
N
Relative mRNAs levels (Normalize to ACTB)
nid-sEVs
aid-sEVs
Angptl8
Fgf21
Hgf
SerpinB1

Supplementary Figure 7. *miR-151* energizes MSCs by targeting KLF9. ***A***: qRT-PCR was performed to detect the expression level of *Klf9* in islets of NCD and HFD mice (n=6/group). ***B***: qRT-PCR was performed to detect the expression level of *Klf9* in islets of nid-sEVs and aid-sEVs administrated mice (n=6/group). ***C***: Graphic representation of the conserved *miR-151* binding motif in the *Klf9* 3′-UTR. Binding of the WT (top) and mutated (bottom) murine *Klf9* 3′-UTR to the *miR-151* seed sequence was assessed in reporter gene experiments. ***D***: Anti-Ago2 RIP was performed in HEK-293T transiently overexpressing *Klf9*, followed by qRT-PCR to detect *miR-151* associated with Ago2 (nonspecific IgG served as NC) (n=3/group). ***E***: The knockdown efficiency of *Klf9* by siRNAs in MSCs (n=3/group). ***F-G***: Primary islets were incubated with supernatant collected from MSCs treated with *Klf9*-siRNA or/and aid-sEVs. GSIS *(F)* and Ki67 staining *(G)* were performed to detect the proliferation and GSIS of islets (n=3/group). For Ki67 staining *(G)*, at least 20 islets were analyzed in each group. Magnification, ×20; scale bar, 20 µm. ***H-I***: The binding motif of KLF9 with Wnts *(H)* and proliferation related genes *(I)* predicted by JASPAR website. ***J-K***: Relative luciferase activity of HEK-293T co-transfected with oe-*Klf9* plasmid and a luciferase reporter containing promoter region of *Wnt3a (J)* or *Ccnd1 (K)*. The data are presented as the relative ratio of Renilla luciferase activity to firefly luciferase activity (n=3/group). ***L-M:*** qRT-PCR was performed to detect the expression levels of *Ccnd1(L)* and *Wnt3a(M)* in MSCs infected with oe-*Klf9* lentivirus (n=3/group). ***N***: qRT-PCR was performed to detect the expression level of *Angptl8*, *Fgf21*, *Hgf* and *SerpinB1* in nid-sEVs and aid-sEVs administrated AML12 cells (n=3/group). Data are presented as mean ± SD. *p<0.05, **p<0.01, ***p<0.001 by Student's t test *(A, B, D, J-N)* or one-way ANOVA *(E-G)*.

Table S1.

Supplementary table 1. Chracteristics of hyperlipidemia samples used in this study

**Characteristics**		**Healthy (n = 11)**	**Hyperlipidemia (n = 16)**
Age (mean±s.d., year)		35.92 ± 10.56 (female: n = 8; male: n = 4)	37.13 ± 6.06 (female: n=2; male: n=14)
Hypercholester-olemia	Total cholesterol (TC) (mean+s.d., mmol/L)	4.13 ± 0.55 (n = 12)	6.08 ± 0.74 (n=11)
Hypertrigly-ceridemia	Total triglyceride (TG) (mean+s.d., mmol/L)	0.99 ± 0.28 (n = 12)	3.12 ± 1.15 (n=14)

A total of 28 human sera were collected for sEVs isolation and NTA analysis, of which 12 were from healthy individuals and 16 were from patients with hyperlipidemia. Patients were classified as having hypercholesterolemia or hypertriglyceridemia if their total cholesterol (TC) exceeds 5.2 mmol/L or their triglyceride (TG) exceeds 1.7 mmol/L.

Table S2.

Supplementary table 2. Chracteristics of hyperglycemia samples used in this study

**Characteristics**	**Healthy (n = 13)**	**Hyperglycemia (n = 14)**
Age (mean±s.d., year)	60.92 ± 9.45 (female: n = 5; male: n = 8)	60.93 ± 13.21 (female: n = 8; male: n = 6)
Fasting blood glucose (mmol/L)	4.88 ± 0.92 (n = 13)	11.44 ± 5.212 (n = 14)

A total of 25 human sera were collected for sEVs isolation and NTA analysis, of which 13 were from healthy individuals and 14 were from patients with hyperglycemia. Patients were classified as having hyperglycemia if their fasting blood glucose (FBG) exceeds 7.0 mmol/L.

Table S3.

Supplementary table 3. membrane proteins in sEVs

Gene Symbol	Intensity
Atp1b1	11734000
Stxbp1	5581300
Scarb2	4153200
Ezr	4065300
Rab10	3394300
Vamp2	3083200
Epb41l3	3078300
Rdx	2773300
Atp6ap1	1659200
Rac1	1653300
Vamp7	1596700
Jup	1461500
F11r	1091900
Pacsin1	860930
Vamp3	446100
Efr3a	326060
Cdh2	316110
Dlg1	286530
Ttc7b	263400
Vamp4	179110
Dpp10	115810
Palm	110550
Hycc1	108680
Sorl1	97758
Gpr158	96889

Tspan5	86765
Golga7	78174
Ptch1	70992
Pdzk1	64141
Wnk3	35220
Pals1	34272
C2cd5	20852
Lama5	18535
Golph3l	15200

The sEVs protein was isolated and subjected to mass spectrometry analysis. The list of proteins obtained from mass spectrometry was intersected with the GO:0072659 gene set “protein localization to plasma membrane,” yielding 34 proteins. These were then sorted in descending order of protein abundance, with intensity representing the measured protein abundance.

Table S4.

Supplementary table 4. sEVs miRNA sequence data

miRNA	sampleA	sampleB	logFC	logCPM	pvalue	FDR	level
mmu-miR-465b-3p	HFD	NCD	11.33474	9.817206	2.66E-13	9.54E-11	Increased
mmu-miR-483-5p	HFD	NCD	9.61117	8.14887	2.14E-09	2.56E-07	Increased
mmu-miR-346-5p	HFD	NCD	9.108095	7.6776	7.39E-08	3.93E-06	Increased
mmu-miR-465c-5p	HFD	NCD	9.091388	7.661303	3.43E-08	2.24E-06	Increased
mmu-miR-743a-3p	HFD	NCD	8.497265	7.118095	7.33E-07	3.09E-05	Increased
mmu-miR-465b-5p	HFD	NCD	8.241993	6.890379	2.45E-06	8.79E-05	Increased
mmu-miR-741-3p	HFD	NCD	7.844271	11.84608	5.18E-14	3.72E-11	Increased
mmu-miR-325-5p	HFD	NCD	7.652573	6.385617	1.22E-04	0.002646	Increased
mmu-miR-384-3p	HFD	NCD	7.582613	6.325441	9.18E-05	0.00206	Increased
mmu-miR-344b-3p	HFD	NCD	7.505789	6.259082	7.13E-05	0.001827	Increased
mmu-miR-880-3p	HFD	NCD	7.490396	6.245485	8.09E-05	0.001874	Increased
mmu-miR-379-3p	HFD	NCD	7.433274	6.200915	1.28E-04	0.002705	Increased
mmu-miR-7688-5p	HFD	NCD	7.326488	6.111933	1.54E-04	0.003118	Increased
mmu-miR-465a-5p	HFD	NCD	7.179479	8.308466	6.63E-09	6.80E-07	Increased
mmu-miR-455-5p	HFD	NCD	7.161168	5.986489	0.001116	0.014845	Increased
mmu-miR-122-5p	HFD	NCD	-4.83521	9.380571	4.68E-07	2.10E-05	Decreased
mmu-miR-296-3p	HFD	NCD	7.155265	8.233771	1.08E-08	9.71E-07	Increased
mmu-miR-325-3p	HFD	NCD	7.067716	9.537482	1.41E-11	2.75E-09	Increased
mmu-miR-329-5p	HFD	NCD	6.881068	5.759475	9.74E-04	0.013447	Increased
mmu-miR-871-3p	HFD	NCD	6.871308	11.62226	4.01E-08	2.40E-06	Increased
mmu-miR-488-3p	HFD	NCD	6.791335	5.685747	0.001838	0.022363	Increased
mmu-miR-470-5p	HFD	NCD	6.790707	10.35326	1.53E-11	2.75E-09	Increased
mmu-miR-15a-3p	HFD	NCD	6.719299	5.636519	0.001535	0.019338	Increased
mmu-miR-344d-3-5p	HFD	NCD	6.683499	5.609114	0.002846	0.030957	Increased
mmu-miR-671-5p	HFD	NCD	6.541694	5.511524	0.002184	0.025296	Increased
mmu-miR-182-3p	HFD	NCD	6.512327	5.485769	0.00467	0.044403	Increased
mmu-miR-344d-3p	HFD	NCD	6.331507	9.502928	7.67E-08	3.93E-06	Increased

mmu-miR-380-3p	HFD	NCD	6.28758	5.331703	0.003423	0.036144	Increased
mmu-miR-743b-3p	HFD	NCD	6.036469	8.165761	2.50E-07	1.20E-05	Increased
mmu-miR-211-5p	HFD	NCD	-7.47032	7.522111	1.96E-04	0.003711	Decreased
mmu-miR-881-3p	HFD	NCD	5.738037	6.932991	8.96E-06	3.06E-04	Increased
mmu-miR-669c-5p	HFD	NCD	5.730155	7.108888	4.74E-05	0.001362	Increased
mmu-miR-124-3p	HFD	NCD	5.50868	10.42544	1.97E-10	2.83E-08	Increased
mmu-miR-2137	HFD	NCD	-3.66912	11.64793	4.49E-04	0.007163	Decreased
mmu-miR-467a-5p	HFD	NCD	5.381335	6.622713	6.98E-05	0.001827	Increased
mmu-miR-673-3p	HFD	NCD	5.364657	6.80436	3.03E-04	0.005433	Increased
mmu-miR-411-3p	HFD	NCD	5.241047	7.71515	2.35E-06	8.79E-05	Increased
mmu-miR-455-3p	HFD	NCD	5.223706	6.482595	0.001012	0.013706	Increased
mmu-miR-672-5p	HFD	NCD	5.122895	8.968962	2.36E-08	1.88E-06	Increased
mmu-miR-196a-5p	HFD	NCD	-5.62322	6.030232	0.001395	0.017887	Decreased
mmu-miR-1983	HFD	NCD	5.091823	7.112168	1.55E-05	5.06E-04	Increased
mmu-miR-224-5p	HFD	NCD	-5.08586	5.394387	0.002076	0.024441	Decreased
mmu-miR-211-3p	HFD	NCD	-7.78771	6.356184	0.002782	0.030787	Decreased
mmu-miR-199b-5p	HFD	NCD	-3.87435	7.044862	0.003087	0.033079	Decreased
mmu-miR-199b-3p	HFD	NCD	-3.48576	8.878083	0.004399	0.043863	Decreased
mmu-miR-150-5p	HFD	NCD	-3.13118	6.881865	0.0047	0.044403	Decreased
mmu-miR-298-5p	HFD	NCD	4.619376	9.774135	2.15E-05	6.70E-04	Increased
mmu-miR-7a-2-3p	HFD	NCD	4.423324	6.520598	6.14E-04	0.009586	Increased
mmu-miR-671-3p	HFD	NCD	4.346097	7.537666	4.13E-04	0.006899	Increased
mmu-miR-374b-5p	HFD	NCD	4.08291	7.930876	2.30E-05	6.87E-04	Increased
mmu-miR-182-5p	HFD	NCD	3.975779	14.2704	3.43E-08	2.24E-06	Increased
mmu-miR-212-5p	HFD	NCD	3.956745	9.299656	7.50E-05	0.001856	Increased
mmu-miR-1193-3p	HFD	NCD	3.847136	6.488996	0.001611	0.019946	Increased
mmu-miR-183-5p	HFD	NCD	3.551635	13.54641	8.19E-07	3.27E-05	Increased
mmu-miR-15b-3p	HFD	NCD	3.484757	6.744617	0.003489	0.036306	Increased
mmu-miR-149-5p	HFD	NCD	3.353606	7.588885	6.80E-04	0.010177	Increased
mmu-miR-362-5p	HFD	NCD	3.193171	7.793594	2.61E-04	0.004805	Increased
mmu-miR-370-3p	HFD	NCD	2.961253	9.442729	7.94E-05	0.001874	Increased

mmu-let-7c-5p	HFD	NCD	2.911062	16.12159	6.85E-05	0.001827	Increased
mmu-miR-411-5p	HFD	NCD	2.891824	8.887956	1.96E-04	0.003711	Increased
mmu-miR-383-5p	HFD	NCD	2.887766	9.342028	3.60E-04	0.006154	Increased
mmu-miR-192-5p	HFD	NCD	2.8372	11.9043	1.56E-04	0.003118	Increased
mmu-miR-484	HFD	NCD	2.621585	10.6385	6.60E-04	0.010078	Increased
mmu-miR-744-5p	HFD	NCD	2.539752	10.12768	4.46E-04	0.007163	Increased
mmu-miR-186-5p	HFD	NCD	2.349213	11.91632	7.29E-04	0.010686	Increased
mmu-miR-700-3p	HFD	NCD	2.306547	10.29616	7.51E-04	0.010788	Increased
mmu-miR-7b-5p	HFD	NCD	2.306135	13.23837	7.89E-04	0.011112	Increased
mmu-let-7d-5p	HFD	NCD	2.226295	11.5312	0.002787	0.030787	Increased
mmu-miR-151-5p	HFD	NCD	2.162006	12.61405	0.00235	0.026781	Increased

sEVs were isolated from islets of NCD and compensated HFD mice and subjected to miRNA sequencing.

Table S5.

Supplementary table 5. Primer sequences

	Sequence (5'-3')
sgDicer-1-F	CACCGAAAGAAAGGACCCATTGGTG
sgDicer-1-R	AAACCACCAATGGGTCCTTTCTTT
sgDicer-2-F	CACCGTGCTGAGGGGCTGCAAAGCA
sgDicer-2-R	AAACTGCTTTGCAGCCCCTCAGCA
sgDicer-3-F	CACCGTGCAGGCCTGCCATGCTGAG
sgDicer-3-R	AAACCTCAGCATGGCAGGCCTGCA
sgDicer-4-F	CACCGGTGAAGGCGATACAGTATGC
sgDicer-4-R	AAACGCATACTGTATCGCCTTCAC
sgSTXBP-1-F	CACCGGCAGTCTCCTGTTACTAGTA
sgSTXBP-1-R	AAACTACTAGTAACAGGAGACTGCC
sgSTXBP-2-F	CACCGGGAGCGGGACCAGAAGTTGT
sgSTXBP-2-R	AAACACAACTTCTGGTCCCGCTCCC
sgSTXBP-3-F	CACCGCACACTCACCTCATCCACCT
sgSTXBP-3-R	AAACAGGTGGATGAGGTGAGTGTGC
sgVAMP2-1-F	CACCGTGTGTCGCAGCGCAATCCAC
sgVAMP2-1-R	AAACGTGGATTGCGCTGCGACACAC
sgVAMP2-2-F	CACCGTGCAGGTGAGTCGAATACTA
sgVAMP2-2-R	AAACTAGTATTCGACTCACCTGCAC
sgVAMP2-3-F	CACCGACCGCACCTGGGACTCATAG
sgVAMP2-3-R	AAACCTATGAGTCCCAGGTGCGGTC
sgF11R-1-F	CACCGTGTTATAACAGCCAGATCAC
sgF11R-1-R	AAACGTGATCTGGCTGTTATAACAC
sgF11R-2-F	CACCGTCTCCTCTCCCCGAGTGGAG
sgF11R-2-R	AAACCTCCACTCGGGGAGAGGAGAC
sgF11R-3-F	CACCGCAAGTGCAGTTGTGCTGCCT
sgF11R-3-R	AAACAGGCAGCACAACTGCACTTGC
Klf9-F	CCGTCGCCCGCACCA

Klf9-R	GTTTCCTGGGAGCTCTGTCC
E2f6-F	CGCGGGACAGCATGAGT
E2f6-R	ACCTTCGTCACTCTGCACTG
Mnt3-F	GGCTGCTCTCGCCGC
Mnt3-R	AGTCAGGTCCCCTTCCCTG

Table S6.

Supplementary table 6. Sequence of *miR-151*

	Sequence (5'-3')
miR-151 mimics sense	UCGAGGAGCUCACAGUCUAGU
miR-151 mimics antisense	UAGACUGUGAGCUCCUCGAUU
miR-151inhibitor	ACUAGACUGUGAGCUCCUCGA

Supplementary data files.

Mass spectrometry results of sEVs.
